# The Etiology of Childhood Pneumonia in The Gambia: Findings From the Pneumonia Etiology Research for Child Health (PERCH) Study

**DOI:** 10.1097/INF.0000000000002766

**Published:** 2021-08-25

**Authors:** Stephen R. C. Howie, Bernard E. Ebruke, Jessica L. McLellan, Maria Deloria Knoll, Michel M. Dione, Daniel R. Feikin, Meredith Haddix, Laura L. Hammitt, Eunice M. Machuka, David R. Murdoch, Katherine L. O’Brien, Ogochukwu Ofordile, Oluyinka E. Olutunde, David Parker, Christine Prosperi, Rasheed A. Salaudeen, Arifin Shamsul, Grant Mackenzie, Martin Antonio, Syed M. A. Zaman

**Affiliations:** From the * Medical Research Council Unit, Basse, The Gambia; † Department of Paediatrics, University of Auckland, New Zealand; ‡ The University of Calgary Cumming School of Medicine, Calgary, Alberta, Canada; § Department of International Health, International Vaccine Access Center, Johns Hopkins Bloomberg School of Public Health, Baltimore, Maryland; ¶ International Livestock Research Institute, Kampala, Uganda; ‖ Department of Pathology, University of Otago, Christchurch, New Zealand; ** Microbiology Unit, Canterbury Health Laboratories, Christchurch, New Zealand; †† AstraZeneca, Cambridge, United Kingdom; ‡‡ Medical Microbiology Department, Lagos University Teaching Hospital, Lagos, Nigeria; §§ Murdoch Children’s Research Institute, Melbourne, Australia; ¶¶ London School of Hygiene & Tropical Medicine; ‖‖ Department of Pathogen Molecular Biology, London School of Hygiene & Tropical Medicine; *** Microbiology and Infection Unit, Warwick Medical School, University of Warwick, Coventry, United Kingdom.

**Keywords:** Gambia, pneumonia, etiology, childhood, Pneumonia Etiology Research for Child Health

## Abstract

Pneumonia remains the leading cause of death in young children globally. The changing epidemiology of pneumonia requires up-to-date data to guide both case management and prevention programs. The Gambia study site contributed a high child mortality, high pneumonia incidence, low HIV prevalence, *Haemophilus influenzae* type b and pneumococcal conjugate vaccines-vaccinated rural West African setting to the Pneumonia Etiology Research for Child Health (PERCH) Study.

**Methods:**

The PERCH study was a 7-country case-control study of the etiology of hospitalized severe pneumonia in children 1–59 months of age in low and middle-income countries. Culture and nucleic acid detection methods were used to test nasopharyngeal/oropharyngeal swabs, blood, induced sputum and, in selected cases, lung or pleural fluid aspirates. Etiology was determined by integrating case and control data from multiple specimens using the PERCH integrated analysis based on Bayesian probabilistic methods.

**Results:**

At The Gambia study site, 638 cases of World Health Organization-defined severe and very severe pneumonia (286 of which were chest radiograph [CXR]-positive and HIV-negative) and 654 age-frequency matched controls were enrolled. Viral causes predominated overall (viral 58% vs. bacterial 28%), and of CXR-positive cases respiratory syncytial virus (RSV) accounted for 37%, *Streptococcus pneumoniae* 13% and parainfluenza was responsible for 9%. Nevertheless, among very severe cases bacterial causes dominated (77% bacterial vs. 11% viral), led by *S. pneumoniae* (41%); *Mycobacterium tuberculosis*, not included in “bacterial”, accounted for 9%. 93% and 80% of controls ≥1 year of age were, respectively, fully vaccinated for age against *Haemophilus influenzae* and *S. pneumoniae*.

**Conclusions:**

Viral causes, notably RSV, predominated in The Gambia overall, but bacterial causes dominated the severest cases. Efforts must continue to prevent disease by optimizing access to existing vaccines, and to develop new vaccines, notably against RSV. A continued emphasis on appropriate case management of severe pneumonia remains important.

## Abstract 2

Supplemental Digital Content is available in the text.

Pneumonia is the biggest single cause of death in children globally, accounting for 15% of 5.3 million deaths in children under the age of 5 years in 2018.^1^ A progress has been made in the reduction of pneumonia morbidity and mortality, but to reach the United Nations Sustainable Development Goal targets for child health, deaths and morbidity from pneumonia will need to be substantially further reduced.^2^ The epidemiology and etiology of pneumonia are changing as a result of global trends strongly influenced by social development and vaccination, principally the roll out of conjugate *Haemophilus influenzae* type b (Hib) and pneumococcal conjugate vaccines (PCV). It is in this context that the Pneumonia Etiology Research for Child Health (PERCH) study has been undertaken to contribute to global pneumonia control efforts for the 21st century. PERCH is a 7-country, 9-site study of childhood pneumonia in 5 countries in Africa (The Gambia, Mali, Kenya, Zambia and South Africa) and 2 countries in Asia (Thailand and Bangladesh).^3^ The overwhelming burden of death and sickness from pneumonia occurs in such low and middle-income countries (LMICs). Specifically, half of childhood deaths from pneumonia occur in sub-Saharan Africa.^4^ The Gambia, like other countries in this region, suffers a high toll from pneumonia, and this report details the etiology of childhood pneumonia in this West African nation with high PCV and Hib vaccine coverage.^5–9^

## STUDY POPULATION AND METHODS

The Gambia, in West Africa, is a resource-poor country with a population of 1.8 million, high child mortality (74 children under 5 per 1000 live births [2012]), low HIV prevalence (<2%) and seasonal malaria transmission.^10, 11^ The Gambia is located within the semiarid Sahel region of sub-Saharan Africa and extends some 400 km inland from the Atlantic coast of West Africa. Childhood pneumonia etiology in The Gambia has been historically dominated by *Streptococcus pneumoniae* and *H. influenzae*, vaccinations for which were introduced in 2009 and 1997 (for Hib), respectively.^12, 13^ The PERCH study site in The Gambia was located in the rural Upper River Region within an area covered by the Basse Health and Demographic Surveillance System (BHDSS),^14^ which is host to ongoing health research including pneumococcal surveillance.^15^ The BHDSS has a population of 178,510 (2014), of which 32,530 (18%) are children under 5 years of age; all births, deaths, pregnancies and migration into and out of the surveillance area are enumerated 3 times a year. The only major health center in the area is the Basse Health Centre, located in Basse township, and the area is also served by several subsidiary smaller health facilities (Supplement Digital Content 1, http://links.lww.com/INF/D996). The Basse site provides the PERCH study representation of a rural, low-HIV-seroprevalence, African setting with seasonal malaria transmission.

### Selection of Participants

We conducted a case-control study comparing severe hospitalized pneumonia cases with community controls. Details of entry criteria for the PERCH study have been described.^3,16^ Cases were children 1–59 months of age with severe pneumonia who presented to government health centers within the BHDSS (Basse, Dembakunda, Garawol and Fatoto Health Centers) between November 3, 2011 and November 2, 2013. We enrolled patients 7 days per week between the hours of 8 am and 6 pm. We defined severe pneumonia using modified World Health Organization (WHO) criteria (2005 definitions),^17^ namely cough or difficulty in breathing, plus “danger signs” to define very severe pneumonia (danger signs comprising central cyanosis, difficulty breast-feeding/drinking, vomiting everything, multiple or prolonged convulsions, lethargy/unconsciousness or head nodding), or lower chest wall indrawing in the absence of danger signs to define severe pneumonia.^16, 18^ We excluded children with wheezing if their lower chest wall indrawing resolved after standardized bronchodilator therapy. Radiologic pneumonia was defined using WHO criteria applied in a standardized interpretation process.^19,20^ Cases were defined as chest radiograph (CXR)-positive if they had either clear consolidation meeting WHO primary endpoint (PEP) criteria or “other infiltrates” not meeting PEP criteria.

Controls comprised children from the community 1–59 months of age randomly selected from within the BHDSS. Potential controls were visited in the community and invited to participate, and those giving informed consent were brought to Basse Health Centre for enrollment procedures. Controls were frequency-matched on age with the goal of a minimum enrollment of 25 controls per month. Those meeting the case definitions for PERCH were excluded, but those with mild acute respiratory infections were not.

### Clinical Samples Collected and Laboratory Analyses Undertaken

Details of clinical samples collected and laboratory testing have been published and are summarized in Supplemental Digital Content 2, http://links.lww.com/INF/D997.^21–23^ In summary, from all participants, we collected nasopharyngeal and oropharyngeal (NP/OP) swabs for polymerase chain reaction (PCR) for respiratory pathogens (FTD Resp-33 kit; Fast-track Diagnostics, Sliema, Malta) and culture (plus serotyping) for pneumococcus, blood for pneumococcal PCR and serum for antibiotic activity testing. From cases, we also collected blood for bacterial culture, HIV testing and malaria parasites, induced sputum for bacterial culture and PCR for respiratory pathogens, gastric aspirates for *Mycobacterium tuberculosis* microscopy and culture in selected cases, and in selected cases meeting strict criteria, we undertook transthoracic percutaneous direct fine needle aspiration of the pneumonia lesion. For PCR, positivity was defined using quantitative PCR density thresholds when that improved distinction between cases and controls, which was the case for the following pathogens with poor specificity (ie, odds ratio [OR] < 1): *S. pneumoniae*, *H. influenzae*, cytomegalovirus (CMV) and *Pneumocystis jirovecii* from NP/OP and *S. pneumoniae* from whole blood.^24–26^

### Statistical Analysis and Data Management

Data were entered, cross-checked and validated using a purpose-built online data management system designed by The Emmes Corporation (Rockville, MD).^27^ Simple descriptive analyses were used to describe the frequency and prevalence of pathogens identified by specific laboratory tests stratified by a range of variables; for specimen results available from cases and controls, ORs, adjusted for age and detection of other pathogens were also calculated. Comparisons between groups were made for categorical variables using logistic regression adjusted for age.

The proportion of pneumonia due to each pathogen was estimated using the PERCH integrated analysis (PIA) method, which is described in detail elsewhere.^28–30^ In brief, the PIA is a Bayesian nested partially latent class analysis that integrates the results for each case from blood culture, NP/OP PCR, whole blood PCR for pneumococcus and induced sputum culture for *M. tuberculosis*. The PIA also integrates test results from controls to account for imperfect test specificity of NP/OP PCR and whole blood PCR. Blood culture results (excluding contaminants) and *M. tuberculosis* results were assumed to be 100% specific (ie, the etiology for a case was attributed 100% to the pathogen that was detected in their blood by culture).

The PIA accounts for imperfect sensitivity of each test/pathogen measurement by using a priori estimates of their sensitivity (ie, estimates regarding the plausibility range of sensitivity which varied by the laboratory test method and pathogen) (see Supplemental Digital Content 3, http://links.lww.com/INF/D998). Sensitivity of blood culture was reduced if blood volume was low (<1.5 mL) or if antibiotics were administered before specimen collection. Sensitivity of NP/OP PCR for *S. pneumoniae* and *H. influenzae* was reduced if antibiotics were administered before specimen collection.

As a Bayesian analysis, both the list of pathogens and their starting “prior” etiologic fraction values were specified a priori, which favored no pathogen over another (ie, “uniform” priors). The pathogens selected for inclusion in the analysis included any noncontaminant bacteria detected by culture in blood at any of the 9 PERCH sites, regardless of whether it was observed at The Gambia site specifically, *M. tuberculosis*, and all of the multiplex quantitative PCR pathogens except those considered invalid because of poor assay specificity (*Klebsiella pneumoniae*^31^ and *Moraxella catarrhalis*). A category called “Pathogens Not Otherwise Specified” was also included to estimate the fraction of pneumonia caused by pathogens not tested for or not observed. A child negative for all pathogens would still be assigned an etiology, which would be either one of the explicitly estimated pathogens (implying a “false negative,” accounting for imperfect sensitivity of certain measurements) or not otherwise specified. The model assumes that each child’s pneumonia was caused by a single pathogen. The model assumes that each child’s pneumonia was caused by a single pathogen.

All analyses were adjusted for age (<1 vs. ≥1 year) to account for differences in pathogen prevalences by this factor. For results stratified by case clinical data (such as by CXR positivity, severity and so on), the test results from all controls were used. However, for analyses stratified by age, only data from controls representative of that age group were used.

The PIA estimated both the individual and population-level etiology probability distributions, each summing to 100% across pathogens where each pathogen has a probability ranging from 0% to 100%. The population-level etiologic fraction estimate for each pathogen was approximately the average of the individual case probabilities and was provided with a 95% credible interval (95% CI), the Bayesian analog of the confidence interval. Primary analyses focused on CXR-positive HIV-negative cases. Descriptive statistical analyses were performed using SAS 9.3 (SAS Institute, Cary, NC); the PIA was performed using the BAKER R package (https://github.com/zhenkewu/baker), R Statistical Software 3.3.1 (The R Development Core Team, Vienna, Austria), and Bayesian inference software JAGS 4.2.0 (http://mcmc-jags.sourceforge.net/).

### Ethics

Bioethical considerations in the PERCH project have been described in detail previously.^32^ We obtained written informed consent for participation in the study from parents or legal guardians of cases and controls. The study was approved by The Gambian Government-Medical Research Council Joint Ethics Committee and the Institutional Review Board of the Johns Hopkins Bloomberg School of Public Health (SCC/EC1062, 3075).

## RESULTS

### Study Participants

**FIGURE 1.**
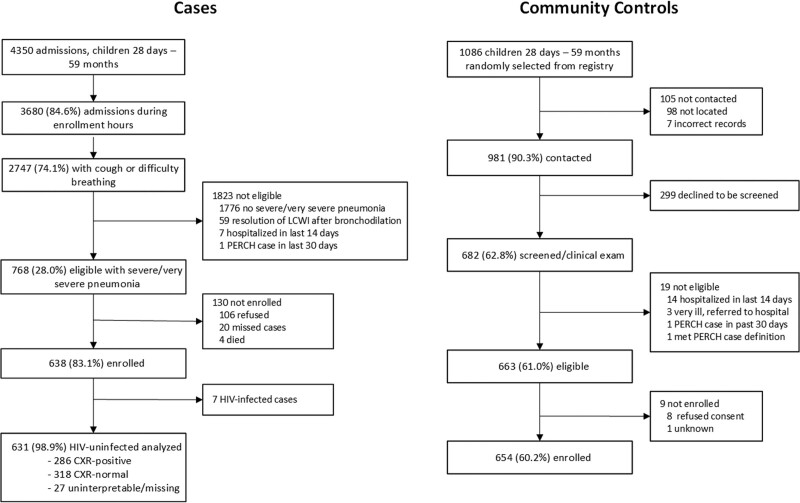
Participant enrollment flow diagram. A: Cases. B: Controls. The number of controls selected for screening is accurate to ±0.3%, reflecting minor inconsistency of recorded numbers.

A total of 638 WHO-defined severe and very severe pneumonia cases (544 [85%] severe and 94 [15%] very severe), 286 (45%) of which were CXR-positive and HIV-negative and 654 community controls were available for analysis (Fig. 1 and Supplementary Digital Content 4, http://links.lww.com/INF/D999). Of those eligible to participate, 85% were enrolled in the study. Seven (1%) cases were HIV-positive and excluded from subsequent analyses.

Demographic and clinical characteristics of the study groups are shown in Table 1. The majority (62.9%) of cases were under 1 year of age. Ten percent of cases had antibiotic exposure before admission and enrollment. Moderate (weight-for-age z score: −2 to −3) or severe (weight-for-age z score: <−3) malnutrition was present in 30% of all cases, 34% of CXR-positive cases and 46% of all very severe cases, and 54% of CXR-positive very severe cases. Half (50.6%) of all cases enrolled had a normal CXR, 16% had consolidation (WHO PEP criteria) and 29% had WHO “other infiltrates.” Hypoxemia was present in 8% of all cases and 12% of CXR-positive cases. Four percent of all cases and 6% of CXR-positive cases died in hospital or within 30 days of admission (following discharge). Among controls ≥1 year of age, 93% were fully vaccinated for age against *H. influenzae* and 80% of controls were fully vaccinated for age against *S. pneumoniae*.

**TABLE 1.** Demographic and Clinical Characteristics of HIV-uninfected Cases and Controls

	All Cases	CXR+ Cases	Controls	CXR+ Severe Cases	CXR+ Very Severe Cases
All	631	286	654	247	39
Age					
Median age (mo) (IQR)	8 (3, 18)	9 (4, 17)	11 (5, 22)	8 (4, 18)	10 (5, 16)
1–5 mo	253 (40.1)	106 (37.1)	199 (30.4)	94 (38.1)	12 (30.8)
6–11 mo	144 (22.8)	71 (24.8)	133 (20.3)	60 (24.3)	11 (28.2)
12–23 mo	136 (21.6)	70 (24.5)	181 (27.7)	59 (23.9)	11 (28.2)
24–59 mo	98 (15.5)	39 (13.6)	141 (21.6)	34 (13.8)	5 (12.8)
Ethnicity					
Wollof	7 (1.1)	3 (1.1)	2 (0.3)	3 (1.2)	0 (0.0)
Mandinka	179 (28.9)	77 (27.3)	165 (25.5)	65 (26.6)	12 (31.6)
Serahule	286 (46.2)	128 (45.4)	258 (39.9)	112 (45.9)	16 (42.1)
Fula	146 (23.6)	74 (26.2)	213 (33.0)	64 (26.2)	10 (26.3)
Other	1 (0.2)	0 (0.0)	8 (1.2)	0 (0.0)	0 (0.0)
Female	243 (38.5)	108 (37.8)	308 (47.1)	90 (36.4)	18 (46.2)
Season of enrollment					
Rainy (June–October)	297 (47.1)	140 (49)	279 (42.7)	125 (50.6)	15 (38.5)
Dry (November–May)	334 (52.9)	146 (51.0)	375 (57.3)	122 (49.4)	24 (61.5)
Respiratory tract illness^a^	—	—	159 (24.3)	—	—
Malaria slide positive	11 (1.9)	3 (1.2)	7 (1.1)	3 (1.3)	0 (0.0)
Pentavalent fully vaccinated for age^b^					
<1 y old	213 (55.6)	96 (56.1)	218 (67.1)	87 (58.4)	9 (40.9)
≥1 y old	202 (93.1)	97 (94.2)	271 (92.8)	83 (94.3)	14 (93.3)
PCV fully vaccinated for age^b^					
<1 y old	211 (55.1)	95 (55.6)	214 (66.0)	86 (57.7)	9 (40.9)
≥1 y old	192 (88.9)	94 (91.3)	232 (80.0)	81 (92.1)	13 (86.7)
Measles fully vaccinated^c^	238 (90.8)	119 (91.5)	303 (89.9)	103 (93.6)	16 (80.0)
Weight-for-age (WHO) z scores					
>−2 z scores	444 (70.4)	188 (65.7)	531 (81.8)	170 (68.8)	18 (46.2)
−3 ≤ z scores ≤ −2	121 (19.2)	64 (22.4)	83 (12.8)	52 (21.1)	12 (30.8)
<−3 z scores	66 (10.5)	34 (11.9)	35 (5.4)	25 (10.1)	9 (23.1)
Weight-for-height (WHO) z scores					
>−2 z scores	459 (73.0)	201 (70.5)	541 (84.3)	179 (72.8)	22 (56.4)
−3 ≤ z scores ≤ −2	114 (18.1)	55 (19.3)	73 (11.4)	46 (18.7)	9 (23.1)
<−3 z scores	56 (8.9)	29 (10.2)	28 (4.4)	21 (8.5)	8 (20.5)
Height-for-age (WHO) z scores					
>−2 z scores	515 (81.6)	234 (81.8)	538 (82.9)	203 (82.2)	31 (79.5)
−3 ≤ z scores ≤ −2	70 (11.1)	34 (11.9)	80 (12.3)	29 (11.7)	5 (12.8)
<−3 z scores	46 (7.3)	18 (6.3)	31 (4.8)	15 (6.1)	3 (7.7)
Antibiotic pretreatment prior to specimen collection^d^	59 (9.5)	30 (10.6)	1 (0.2)	24 (9.8)	6 (15.4)
Serum antibiotic activity	42 (7.3)	20 (7.6)	1 (0.2)	16 (7.0)	4 (11.4)
CXR result					
Any abnormality	286 (45.3)	286 (100)	—	247 (100)	39 (100)
Any consolidation	101 (16.1)	101 (35.3)	—	80 (32.4)	21 (53.8)
Other infiltrate only	185 (29.4)	185 (64.7)	—	167 (67.6)	18 (46.2)
Normal	318 (50.6)	0 (0.0)	—	0 (0.0)	0 (0.0)
Uninterpretable	25 (4.0)	0 (0.0)	—	0 (0.0)	0 (0.0)
Hypoxemia^e^	49 (7.8)	33 (11.5)	—	23 (9.3)	10 (25.6)
Tachypnea^f^	547 (86.7)	260 (90.9)	—	227 (91.9)	33 (84.6)
Tachycardia^g^	342 (54.3)	159 (55.8)	—	138 (56.1)	21 (53.8)
Very severe pneumonia^h^	94 (14.9)	39 (13.6)	—	0 (0.0)	39 (100)
Danger signs					
Head nodding	28 (4.4)	17 (5.9)	—	0 (0.0)	17 (43.6)
Central cyanosis	4 (0.6)	4 (1.4)	—	0 (0.0)	4 (10.3)
Convulsions^i^	29 (4.6)	5 (1.7)	—	0 (0.0)	5 (12.8)
Lethargy^j^	55 (8.7)	23 (8)	—	0 (0.0)	23 (59.0)
Unable feed	17 (2.7)	6 (2.1)	—	0 (0.0)	6 (15.4)
Vomiting	8 (1.3)	2 (0.7)	—	0 (0.0)	2 (5.1)
Crackles	486 (77.1)	231 (81.1)	—	200 (81.3)	31 (79.5)
Wheeze on auscultation	198 (31.5)	84 (29.6)	—	76 (31.0)	8 (20.5)
Grunting	50 (7.9)	26 (9.1)	—	16 (6.5)	10 (25.6)
Nasal flaring	332 (52.6)	161 (56.3)	—	133 (53.8)	28 (71.8)
Elevated temperature (≥38°C)	205 (32.5)	117 (40.9)	—	98 (39.7)	19 (48.7)
Leukocytosis^k^	196 (43.1)	100 (49.8)	—	81 (45.8)	19 (79.2)
CRP ≥ 40 mg/L	132 (34.5)	86 (48.9)	—	73 (47.7)	13 (56.5)
Severe anemia (0–7.5 g/dL)	33 (7.3)	14 (7.0)	—	9 (5.1)	5 (20.8)
Duration of illness^l^					
Median duration of illness (d)^l^ (IQR)	3 (2, 3)	3 (2, 4)	—	3 (2, 4)	3 (2, 5)
0–2 d	256 (40.6)	112 (39.2)	—	98 (39.7)	14 (35.9)
3–5 d	332 (52.6)	149 (52.1)	—	130 (52.6)	19 (48.7)
>5 d	43 (6.8)	25 (8.7)	—	19 (7.7)	6 (15.4)
Median duration of hospitalization (d) (IQR)	3 (2, 4)	3 (2, 4)	—	3 (2, 4)	4 (2, 5)
Duration of hospitalization					
0–2 d	15 (2.4)	9 (3.2)	—	84 (34.3)	10 (25.6)
3–5 d	554 (88.5)	239 (84.2)	—	134 (54.7)	20 (51.3)
>5 d	57 (9.1)	36 (12.7)	—	27 (11.0)	9 (23.1)
Died in hospital or within 30 d of admission	27 (4.3)	16 (5.6)	—	5 (2.0)	11 (28.2)
Died in-hospital	21 (3.3)	12 (4.2)	—	2 (0.8)	10 (25.6)
Died postdischarge, within 30 d of admission^m^	6 (1.0)	4 (1.5)	—	3 (1.2)	1 (3.5)
Missing 30-d vital status^m^	6 (1.0)	2 (0.7)	—	1 (0.4)	1 (3.5)
IQR indicates interquartile range.					

^a^Controls were considered to have respiratory tract illness (RTI) if they had (1) cough (observed or reported) or runny nose (reported) or (2) one of the following: ear discharge (reported), wheeze (reported) or difficulty breathing (reported), in the presence of sore throat (reported) or fever (observed temperature ≥38.0°C or reported fever in the past 48 hours).

^b^For children <1 year, defined as received at least one dose and up-to-date for age based on the child’s age at enrollment, doses received and country schedule (allowing 4-week window each for dose). For children > 1 year, defined as 3+ doses.

^c^At least 1 dose, restricted to children >10 months.

^d^Defined as serum bioassay positive (cases and controls), antibiotics administered at the referral facility, or antibiotic administration prior to whole-blood specimen collection at the study facility (cases only).

^e^Hypoxemia defined as oxygen saturation <92%, or on supplemental oxygen if a room air oxygen saturation reading was not available. A room air oxygen saturation reading was available from 616 (97.6%) of children.

^f^Tachypnea defined as ≥60 breaths per minute (<2 months), ≥50 breaths per minute (2–11 months) and ≥40 breaths per minute (12–59 months).

^g^Tachycardia defined as >160 beats per minute (bpm) (<11 months), >150 bpm (12–35 months) and >140 bpm (36–59 months).

^h^Very severe pneumonia defined as cough or difficulty breathing, and at least one of the following: central cyanosis, difficulty breast-feeding/drinking, vomiting everything, convulsions, lethargy, unconsciousness or head nodding.

^i^Multiple or prolonged convulsions (≥15 min).

^j^Lethargic or unresponsive (responds to voice or pain, unresponsive or pharmacologically sedated).

^k^Leukocytosis count defined as >15 × 10^9^ cells/L for children 1–11 months and >13 × 10^9^ cells/L for children 12–59 months.

^l^Defined as maximum days of reported symptoms for any of the following: cough, wheeze, fever or difficulty breathing.

^m^Restricted to those children discharged alive.

Positive blood cultures were observed in 14 (5%) of CXR-positive cases: 7 (50%) were *S. pneumoniae* (n = 2 PCV13 serotypes and n = 5 non-PCV13 serotypes), 2 were *H. influenzae* non-type b and the remaining were single occurrences of other pathogens (Table 2). Lung aspirates were available from 21 CXR-positive cases with *S. pneumoniae*, *M. catarrhalis* and *H. influenzae* being the most commonly detected pathogens, while the single pleural aspirate sample cultured *Staphylococcus aureus* (Table 2). There were 6/13 (46%) confirmed pneumococcal pneumonia cases with vaccine-type serotypes (n = 3 ST1 and n = 1 each ST5, ST19F and ST6A) (Supplemental Digital Content 5, http://links.lww.com/INF/D1000). *M. tuberculosis* was cultured from the induced sputum of 7 (2.7%) CXR-positive cases (Table 2). The prevalence and ORs for pathogens detected on NP/OP in CXR-positive cases and controls are shown in supplemental digital content 6, http://links.lww.com/INF/E2; 7, http://links.lww.com/INF/E3 and 8, http://links.lww.com/INF/E4. Positivity was significantly associated with case status for 6 pathogens (*S. pneumoniae* [threshold], HMPV A/B, influenza A, parainfluenza 1 and 3, CMV and respiratory syncytial virus [RSV]).

**TABLE 2.** Detection of Organisms in Case-only Specimens, by Specimen and Test

Organism	Test	All Cases	CXR+ Cases
A. Blood		**N = 615**	**N = 279**
Any^a^	Culture	26 (4.2)	14 (5.0)
* Streptococcus pneumoniae*	Culture	10 (1.6)	7 (2.5)
*S. pneumoniae* VT (PCV13)	Culture	2 (0.3)	2 (0.7)
*S. pneumoniae* non-VT (PCV13)	Culture	8 (1.3)	5 (1.8)
* Haemophilus influenzae*	Culture	3 (0.5)	2 (0.7)
*H. influenzae* type b	Culture	1 (0.2)	0 (0.0)
*H. influenzae* non-type b	Culture	2 (0.3)	2 (0.7)
*Staphylococcus aureus*	Culture	3 (0.5)	1 (0.4)
* Salmonella* spp.^b^	Culture	2 (0.3)	1 (0.4)
Other nonfermentative Gram-negative rods^c^	Culture	1 (0.2)	0 (0.0)
Enterobacteriaceae^d^	Culture	4 (0.7)	1 (0.4)
* Neisseria meningitides*	Culture	2 (0.3)	1 (0.4)
* Candida* sp.	Culture	1 (0.2)	1 (0.4)
Mixed^e^	Culture	1 (0.2)	0 (0.0)
Contaminants	Culture	43 (7.0)	24 (8.6)
**B. Lung aspirate** ^f^			
		**N = 22**	**N = 21**
Any	Culture	5 (22.7)	5 (23.8)
	PCR	6 (35.3)	6 (35.3)
	Culture or PCR	8 (36.4)	8 (38.1)
* S. pneumoniae*	Culture	5 (22.7)	5 (23.8)
	PCR	5 (29.4)	5 (29.4)
	Culture or PCR	7 (31.8)	7 (33.3)
*S. pneumoniae* VT (PCV13)^g^	Culture or PCR	4 (18.2)	4 (19.2)
*S. pneumoniae* non-VT (PCV13)^g^	Culture or PCR	2 (9.1)	2 (9.5)
* Moraxella Catarrhalis*	Culture	0 (0.0)	0 (0.0)
	PCR	3 (17.6)	3 (17.6)
	Culture or PCR	3 (13.6)	3 (14.3)
* H. influenzae* ^h^	Culture	1 (4.5)	1 (4.8)
	PCR	2 (11.8)	2 (11.8)
	Culture or PCR	2 (9.1)	2 (9.5)
**C. Pleural fluid** ^f,i^			
		**N = 1**	**N = 1**
Any	Culture	1 (100)	1 (100)
* S. aureus*	Culture	1 (100)	1 (100)
**D. Induced sputum**			
		**N = 575**	**N = 255**
* Mycobacterium tuberculosis*	Culture	7 (1.2)	7 (2.7)

^a^Excludes contaminants.

^b^Nontyphoidal *Salmonella* species (N = 2).

^c^Acinetobacter species (N = 1).

^d^*Enterobacter cloacae* (N = 1); *Escherichia coli* (N = 3); *Klebsiella pneumoniae* (N = 1).

^e^*E. coli* and *K. pneumoniae* (N = 1).

^f^Restricted to specimens obtained within 3 days of admission and those pathogens determined by the clinical review team to be noncontaminants. Data reflect any positivity; some children were positive for multiple organisms on lung aspirate. 22/22 cases had lung aspirate culture results available, and 17/22 had lung aspirate PCR results available. Denominator for “Culture or PCR” rows reflects children with either culture or PCR (N = 22).

^g^Two cases were positive for *S. pneumoniae* by lung aspirate PCR only so no lung aspirate serotype data available. For one of these cases, the serotype detected on induced sputum (ST = 1) was assumed to the ST that would have been detected on lung aspirate because it is rarely detected in healthy children. One case was positive for an *S. pneumoniae* non-PCV13 type serotype on both blood culture and lung aspirate culture.

^h^One case was positive for *H. influenzae* by culture and PCR; serotyping results for culture isolate were missing but were *H. influenzae* non-type b by PCR. One case positive for *H. influenzae* type b by PCR.

^i^One case with a pleural fluid specimen was missing pleural fluid PCR results.

### Seasonality

**FIGURE 2.**
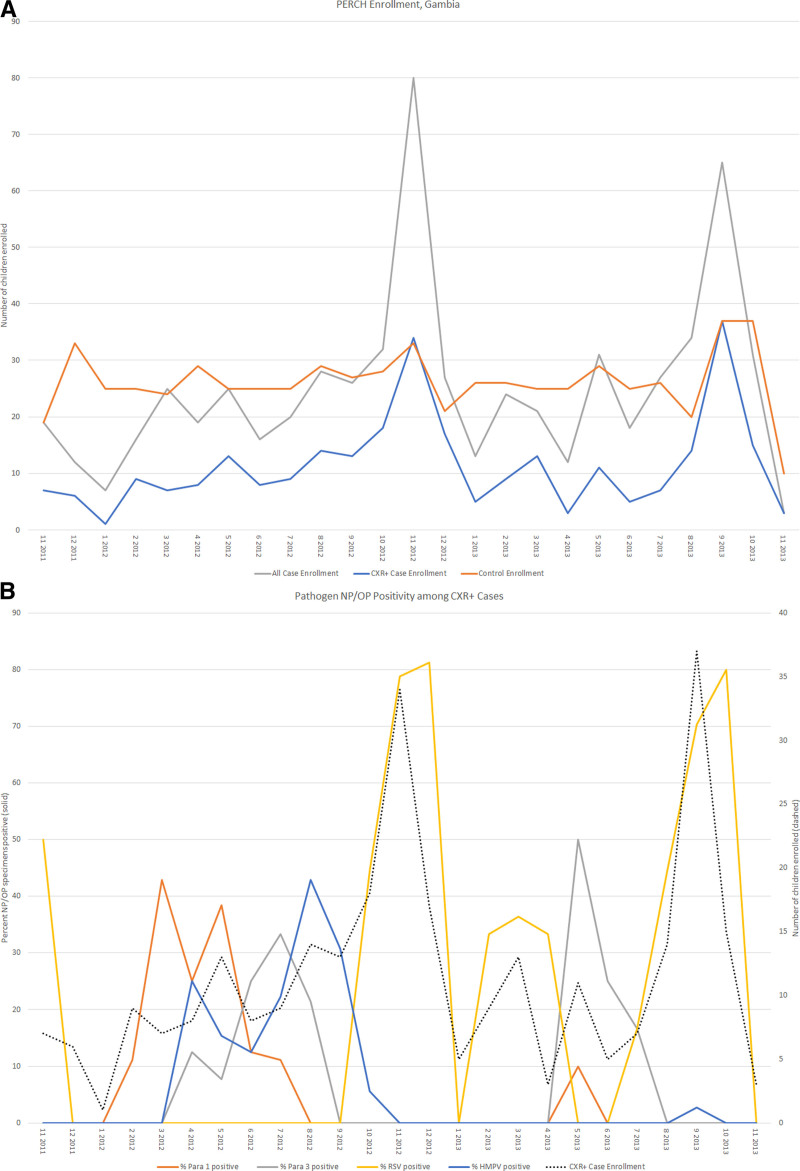
Enrollment and NP/OP PCR positivity by month of study. A: PERCH enrollment by month, all cases, controls, and CXR+ cases. CXR+ defined as consolidation and/or other infiltrate on chest radiograph (subset of the all case groups).B: Enrollment and NP/OP PCR positivity for select viruses by month, CXR+ cases. CXR+ defined as consolidation and/or other infiltrate on chest radiograph. HMPV, human metapneumovirus A/B; NP/OP, nasopharyngeal/oropharyngeal; Para 1, parainfluenza virus types 1; Para 3, para, parainfluenza virus types 3; RSV, respiratory syncytial virus A/B.

The frequency of all case admissions and CXR-positive admissions was markedly seasonal, with a 3- to 4-fold higher peak in the latter part of the calendar year (Fig. 2A). The peaks in CXR-positive cases corresponded closely with an increased prevalence of NP/OP PCR positivity for respiratory viruses, notably RSV and parainfluenza (Fig. 2B).

### Etiology

**FIGURE 3.**
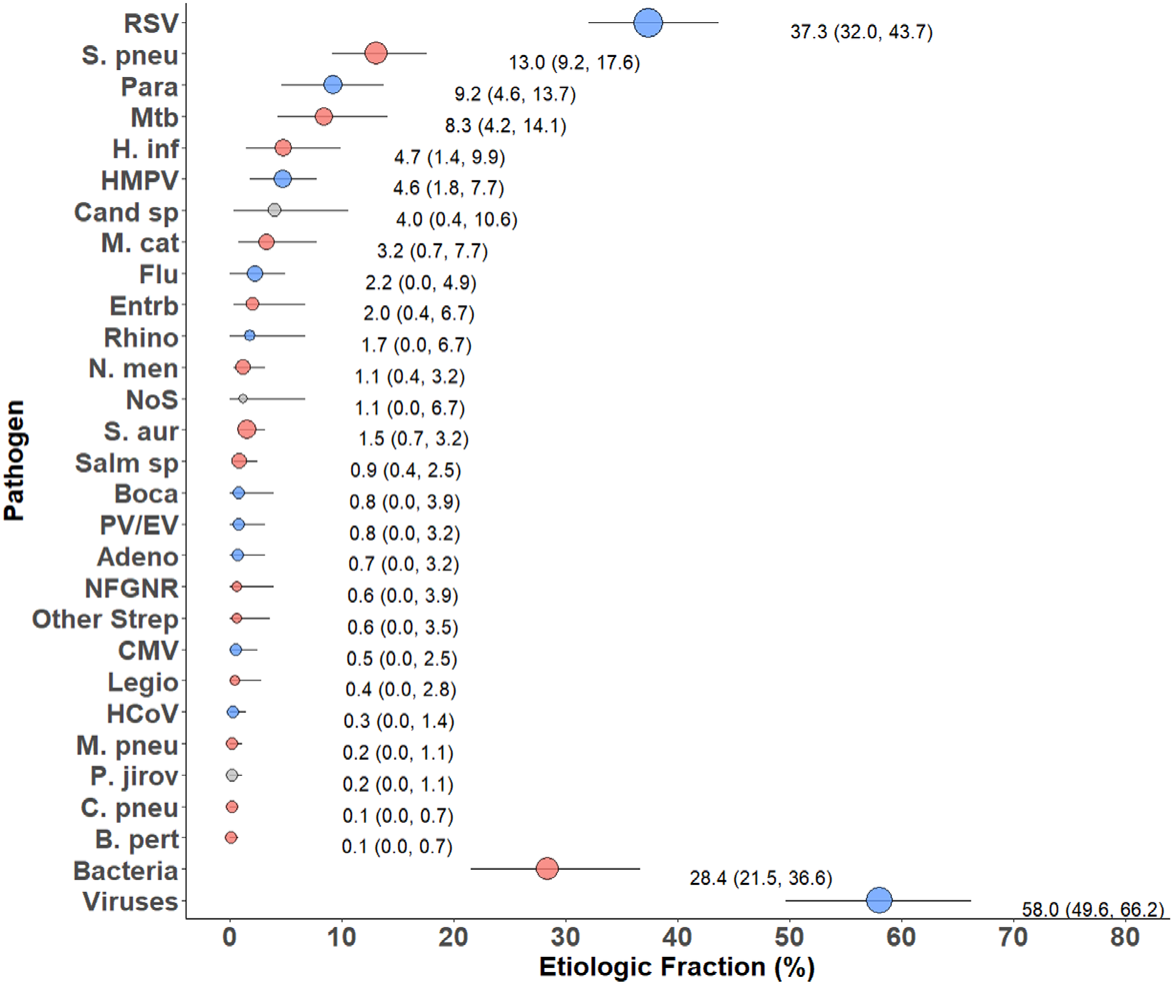
Integrated etiology results, CXR+ cases. Other Strep includes *Streptococcus pyogenes* and *Enterococcus faecium*. NFGNR includes *Acinetobacter* species and *Pseudomonas* species. Enterobacteriaceae includes *Escherichia coli*, *Enterobacter* species and *Klebsiella* species, excluding mixed Gram-negative rods. CXR+ defined as consolidation and/or other infiltrate on chest radiograph. Bacterial summary excludes Mtb. Pathogens that were estimated at the subspecies level but grouped to the species level for display include Parainfluenza virus type 1, 2, 3 and 4; *S. pneumoniae* PCV 13 and *S. pneumoniae* non-PCV 13 types; *H. influenzae* type b and *H. influenzae* non-type b; and influenza A, B and C. Etiologic fraction estimates, including subspecies and serotype disaggregation (eg, PCV-13 type and non-PCV-13 type), are given in Supplemental Digital Content 6, http://links.lww.com/INF/E2. Line represents the 95% credible interval. The size of the symbol is scaled on the basis of the ratio of the estimated etiologic fraction to its standard error. Of 2 identical etiologic fraction estimates, the estimate associated with a larger symbol is more informed by the data than the priors. Adeno indicates adenovirus; B. pert, *Bordetella pertussis*; Boca, human bocavirus; C. pneu, *Chlamydophila pneumoniae*; Cand sp., *Candida* species; CMV, cytomegalovirus; Entrb, enterobacteriaceae; Flu, influenza virus A, B and C; H. inf, *Haemophilus influenzae*; HCoV, coronavirus; HMPV, human metapneumovirus A/B; Legio, *Legionella* species; M. cat, *Moraxella catarrhalis*; M. pneu, *Mycoplasma pneumoniae*; Mtb, *Mycobacterium tuberculosis*; NFGNR, nonfermentative Gram-negative rods; N. men, *Neisseria meningitidis*; NoS, not otherwise specified (ie, pathogens not tested for); P. jirov, *P. jirovecii*; Para, parainfluenza virus types 1, 2, 3 and 4; PV/EV, parechovirus/enterovirus; Rhino, human rhinovirus; RSV, respiratory syncytial virus A/B; S. aur, *Staphylococcus aureus*; S. pneu, *Streptococcus pneumoniae*; Salm sp, *Salmonella* species.

**FIGURE 4.**
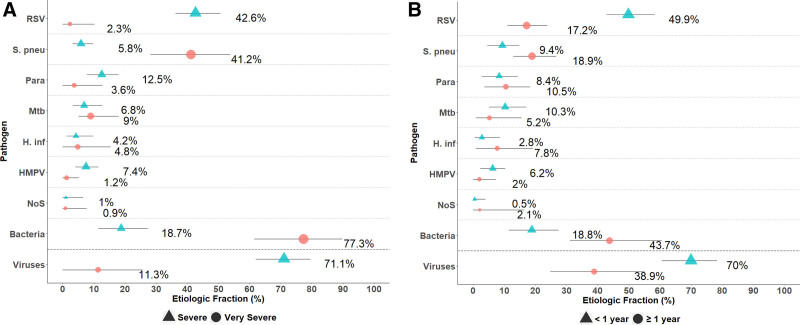
Integrated etiology results, CXR+ cases, by (A) pneumonia severity and (B) age, selected pathogens. CXR+ defined as consolidation and/or other infiltrate on chest radiograph. Bacterial summary excludes Mtb. Very severe pneumonia defined as cough or difficulty breathing, and at least one of the following: central cyanosis, difficulty breast-feeding/drinking, vomiting everything, convulsions, lethargy, unconsciousness or head nodding. Sample sizes: CXR+ severe, N = 247; CXR+ very severe, N = 39. CXR+ <1 year, N = 177; CXR+ ≥1 year, N = 109. Pathogens that were estimated at the subspecies level but grouped to the species level for display include Parainfluenza virus types 1, 2, 3 and 4; *S. pneumoniae* PCV 10 and *S. pneumoniae* non-PCV 10 types; and *H. influenzae* type b and *H. influenzae* non-type b. Line represents the 95% credible interval. The size of the symbol is scaled based on the ratio of the estimated etiologic fraction to its standard error. Of two identical etiologic fraction estimates, the estimate associated with a larger symbol is more informed by the data than the priors. H. inf indicates *Haemophilus influenzae*; HMPV, human metapneumovirus A/B; Mtb, *Mycobacterium tuberculosis*; NoS, not otherwise specified (ie, pathogens not tested for); Parainfluenza virus types 1, 2, 3 and 4; Respiratory syncytial virus A/B; S. pneu, *Streptococcus pneumoniae*.

Among CXR-positive cases RSV (37.3%) and *S. pneumoniae* (13.0%) were the leading causes, followed by parainfluenza viruses (9.2%), *M. tuberculosis* (8.3%) and *H. influenzae* (4.7%), with viruses accounting for 58% of cases and bacteria 28% (Fig. 3, Supplemental Digital Content 9, http://links.lww.com/INF/E5). The leading causes of severe (but not very severe) CXR-positive pneumonia were, similarly, RSV and parainfluenza viruses, with 71% of cases estimated to have a viral etiology (Fig. 4A, Supplemental Digital Content 10, http://links.lww.com/INF/E6). In contrast, in CXR-positive very severe pneumonia cases, bacterial causes dominated (76.8%) with *S. pneumoniae* (41.2%) being the leading cause (Fig. 4A and Supplemental Digital Content 10, http://links.lww.com/INF/E6).

In those with CXR-positive severe or very severe pneumonia, the leading cause in older children (1 or more years of age) was *S. pneumoniae* followed by RSV (Fig. 4B and Supplemental Digital Content 11, http://links.lww.com/INF/E7), while the leading cause in younger infants under 1 year of age was RSV (49.9%) followed by *M. tuberculosis* (10.3%), *S. pneumoniae* (9.4%) and parainfluenza (8.4%); similar findings by age were seen when restricting to severe pneumonia cases (Supplemental Digital Content 12, http://links.lww.com/INF/E8). Findings from CXR-positive cases were similar to those from all cases, which showed that RSV and parainfluenza dominated, accounting for half (49.8%) of pneumonia etiologies as estimated by the PIA method, with viral causes responsible for 66% overall, bacterial causes 26.2% and *M. tuberculosis* 5.1% (Supplemental Digital Content 9, http://links.lww.com/INF/E5 and 13, http://links.lww.com/INF/E9).

Etiology differed by malnutrition status, with bacterial infection being more prevalent in acutely malnourished children (53.7% in those with a weight-for-height z-score below −2 vs. 29.1% in those with a weight-for-height z-score above than −2); specifically, RSV was less prevalent in the undernourished (19.8% vs. 37.0%), while *H. influenzae* (13.7% vs. 3.1%) and Enterobacteriaceae (4.2% vs. 0.4%) were more prevalent in the undernourished (data not shown). There was one blood culture positive for *Candida* species in a severely chronically malnourished child 20 months of age. This one positive translated into an etiology fraction of 4.0% for *Candida* species among CXR-positive cases, with sensitivity analyses around the sensitivity of blood culture for *Candida* indicating that it is unlikely to be less than 2%.

In-hospital case fatality rates were 3.3% overall, 4.2% in CXR-positive cases, 19.1% in all very severe cases and 25.6% in very severe CXR-positive cases. Among the 21 deaths, bacterial causes were prominent (8 normally sterile sample-confirmed; Supplemental Digital Content 14, http://links.lww.com/INF/E10).

## DISCUSSION

This study showed that the leading causes of severe pneumonia in young children in The Gambia site were RSV, *S. pneumoniae*, parainfluenza and *M. tuberculosis*, and in very severe cases, bacterial causes predominated, notably *S. pneumoniae*. RSV was the leading pathogen in the PERCH study overall, and the findings in The Gambia site reinforce its importance in all subgroups, and notably in the youngest children.^29^ The priority of developing interventions to reduce the RSV-pneumonia burden is high, as previously discussed.^29^ The Gambia had a higher prevalence of parainfluenza than other PERCH study sites, the result of more than 1 outbreak during the study period and a reminder that epidemic pathogens are an ever-present threat.

The Upper River Region study site in The Gambia was selected for participation in the PERCH Study because of its rural West African location. It complements the urban West African site in Mali, the urban East African site in Zambia, the rural East African site in Kenya and the Asian sites in Thailand and Bangladesh. It is notable that The Gambia has a low gross domestic product per capita, a high child mortality rate, seasonal malaria transmission and a low HIV infection prevalence.^10,11^

While viral causes such as RSV and parainfluenza predominated overall, pneumococcus was the leading pathogen among the very severe pneumonia cases, even in the presence of high PCV coverage, and bacterial causes accounted for three-quarters of very severe cases. The continued importance of bacterial pathogens in very severe cases and in deaths continues to support the priority of prompt treatment of cases with appropriate antibiotics in countries like The Gambia and the continued development and increased use of bacterial-target vaccines.

In a previous pre-PCV (but Hib vaccine era) study of lung aspirate samples from children with CXR-positive severe pneumonia, bacterial causes dominated, led by *S. pneumoniae* and followed by *H. influenzae*, mostly nontypeable.^33^ This is consistent with previous studies of severe pneumonia in The Gambia with the exception that pre-Hib vaccination most *H. influenzae* was type b. The *H. influenzae* identified in the current study was mostly non-type b, which is consistent with the very low rate of Hib infection seen in the Gambian surveillance studies.^34^ Of the confirmed pneumococcal pneumonia cases, 46% were vaccine-type; while this study was not designed to assess vaccine effectiveness the finding reinforces the importance of ongoing surveillance to monitor this.

It is notable that tuberculosis played an important role in pneumonia etiology in this study. Previous studies of radiologic childhood pneumonia in The Gambia in the pre-PCV and pre-Hib vaccine era identified tuberculosis in 1%–4% of cases. In PERCH, tuberculosis was responsible for an estimated 8.3% of CXR-positive pneumonia cases in The Gambia.^35,36^ Tuberculosis should be in clinicians’ minds, especially where it is prevalent and where children do not respond adequately to empirical treatment with standard antibiotics.^37^

This study highlights the importance of epidemic pathogens such as parainfluenza viruses, notably types 1 and 3, which featured strongly in The Gambia.^29^ Parainfluenza epidemics occur periodically and can cause considerable morbidity in children, typically lower respiratory tract infections in infants and laryngotracheobronchitis (croup) in preschoolers.^38,39^ It is notable that the burden of parainfluenza-associated pneumonia in The Gambia was not the result of a single outbreak but several from more than 1 serotype. While efforts toward the development and application of respiratory viral vaccines have focused historically on measles and influenza, and more recently RSV, future efforts may need to add parainfluenza.^40^

Severe childhood pneumonia in The Gambia occurs year-round; nevertheless, important seasonal differences were observed in pneumonia incidence and etiology, with the “pneumonia season” typically occurring between September and November annually, which coincides with the latter part of the hot humid rainy season. In the PERCH study, these peaks coincided with outbreaks of RSV (which occur annually).

This study had the strengths of a carefully designed and executed case-control study, the characteristics of which have been well described.^20–23,27^ Standardization of methods and sufficient power at The Gambian site lend weight to the findings reported here. Eighty-five percent of all admissions occurred during enrollment hours; however, because overnight admissions were excluded, it is possible that enrollment bias against more severe or fatal cases presenting after hours may have resulted. There are, additionally, well-described limitations of any case-control study, even a large one, and the scarcity of gold-standard specimens, such as lung and pleural aspirates, make a degree of caution in interpretation of the findings appropriate. There is also the limitation of the WHO diagnostic criteria, which are sensitive but not specific for pneumonia, meaning that some cases without pneumonia, for instance with bronchiolitis or sepsis, will be categorized as pneumonia. Nevertheless, the similarity of results between those with CXR-positive pneumonia criteria and those with WHO clinically defined pneumonia argues against important bias resulting from case definitions.

Although PERCH relied on multiple samples (blood, NP/OP swabs, induced sputum) to estimate etiology, they were not taken directly from the site of infection (the lung), which is true for most etiology studies. Nevertheless, these specimens do have diagnostic potential, as demonstrated by association with case status within the PERCH analysis, and indeed in standard clinical practice, such as NP/OP PCR results for pertussis and RSV or induced sputum for tuberculosis. Unfortunately, there is no fool-proof method for distinguishing chronic carriage from newly acquired exposure or infection in a case control study; however, using quantitative PCR and applying density thresholds that were associated with detection in the blood improved the value of the data. The PIA model assumes that each case’s pneumonia episode is caused by a single pathogen, and it does not attempt to identify or quantify pathogen combinations. While we acknowledge that copathogen causes will result in an underestimate of any single cause, exploratory analyses do not support a large underestimate (data not shown). Last, it is important to acknowledge that, within the broad methodologic challenges facing pneumonia etiology studies, the PIA model takes an important step forward in accounting for and mitigating those limitations, and this is an important contribution the PERCH study makes to the field.

## CONCLUSIONS

At The Gambian site of the PERCH study, viral causes predominated overall, as they did in other sites, with RSV being the leading cause. *S. pneumoniae* was the second most prevalent cause in The Gambia, followed by Parainfluenza, the result of several outbreaks during the study period, and a reminder of the importance of epidemic pathogens. Tuberculosis was also a notable cause, highlighting that the tuberculosis global pandemic continues to impact children. Despite the preponderance of viral causes overall, very severe cases were predominantly bacterial, with *S. pneumoniae* most important despite high coverage with PCV immunization in the population. These findings support the continued emphasis on appropriate case management of severe pneumonia and also ongoing efforts to prevent disease by developing new vaccines, notably to RSV, and optimizing access to existing vaccines.

## ACKNOWLEDGMENTS

We offer sincere thanks to the children and families who participated in this study and the staff of The Gambian Ministry of Health, who contributed so much to the study. We would also like to acknowledge members of the following groups who contributed to the study design, conduct and analysis phases of PERCH: Pneumonia Methods Working Group, PERCH Expert Group, PERCH Chest Radiograph Reading Panel and Shalika Jayawardena and Rose Watt from Canterbury Health Laboratories. We also acknowledge the substantial contributions of the other members of the PERCH Study Group not listed as coauthors (see below).

Johns Hopkins Bloomberg School of Public Health, Baltimore, Maryland: Orin S. Levine (Former PI, current affiliation Bill & Melinda Gates Foundation, Seattle, Washington), Andrea N. DeLuca, Amanda J. Driscoll, Nicholas Fancourt, Wei Fu, Melissa M. Higdon, E. Wangeci Kagucia, Ruth A. Karron, Mengying Li, Daniel E. Park, Qiyuan Shi, Zhenke Wu, Scott L. Zeger; The Emmes Corporation, Rockville, Maryland: Nora L. Watson; Nuffield Department of Clinical Medicine, University of Oxford, United Kingdom: Jane Crawley; University of Otago, Christchurch, New Zealand: David R. Murdoch; KEMRI-Wellcome Trust Research Programme, Kilifi, Kenya: J. Anthony G. Scott (site PI and PERCH co-PI, joint affiliation with London School of Hygiene and Tropical Medicine, London, UK); Division of Infectious Disease and Tropical Pediatrics, Department of Pediatrics, Center for Vaccine Development, University of Maryland School of Medicine, Baltimore, Maryland and Centre pour le Développement des Vaccins (CVD-Mali), Bamako, Mali: Karen L. Kotloff (site PI); Medical Research Council: Respiratory and Meningeal Pathogens Research Unit and Department of Science and Technology/National Research Foundation: Vaccine Preventable Diseases, University of the Witwatersrand, Johannesburg, South Africa: Shabir A. Madhi (site PI); Thailand Ministry of Public Health – U.S. CDC Collaboration, Nonthaburi, Thailand: Henry C. Baggett (site PI), Susan A. Maloney (former site PI); Boston University School of Public Health, Boston, Massachusetts and University Teaching Hospital, Lusaka, Zambia: Donald M. Thea (site PI); International Centre for Diarrhoeal Disease Research, Bangladesh (ICDDR, b): W. Abdullah Brooks (site PI); Canterbury Health Laboratories, Christchurch, New Zealand: Trevor P. Anderson, Joanne Mitchell, Shalika Jayawardena, Rose Watt.

## Supplementary Material


