# Supplementary material for: The Etiology of Childhood Pneumonia in The Gambia: Findings From the Pneumonia Etiology Research for Child Health (PERCH) Study
Source: Pediatr Infect Dis J. 2021 Aug 25;40(9):S7–S17. doi: 10.1097/INF.0000000000002766 (PMC8448408; doi:10.1097/INF.0000000000002766)
Supplement: Supplementary file 1 [file inf-40-s07-s001.docx]

**Supplemental Digital Content 1, Figure: Map of The Gambia, showing the Basse Health and Demographic Surveillance Site (BHDSS) study area, along with health facilities and population settlements**


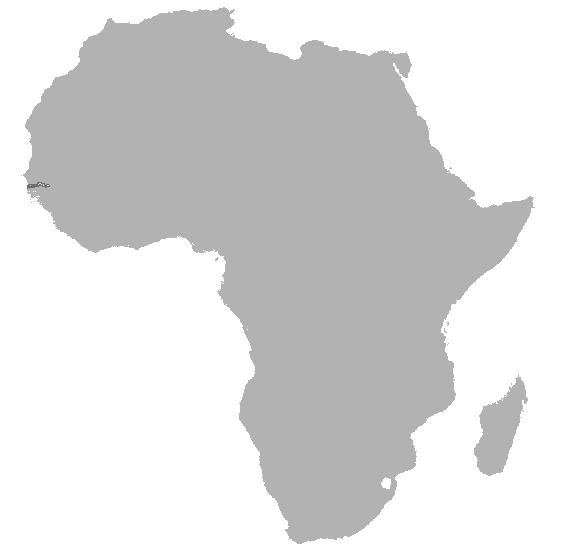

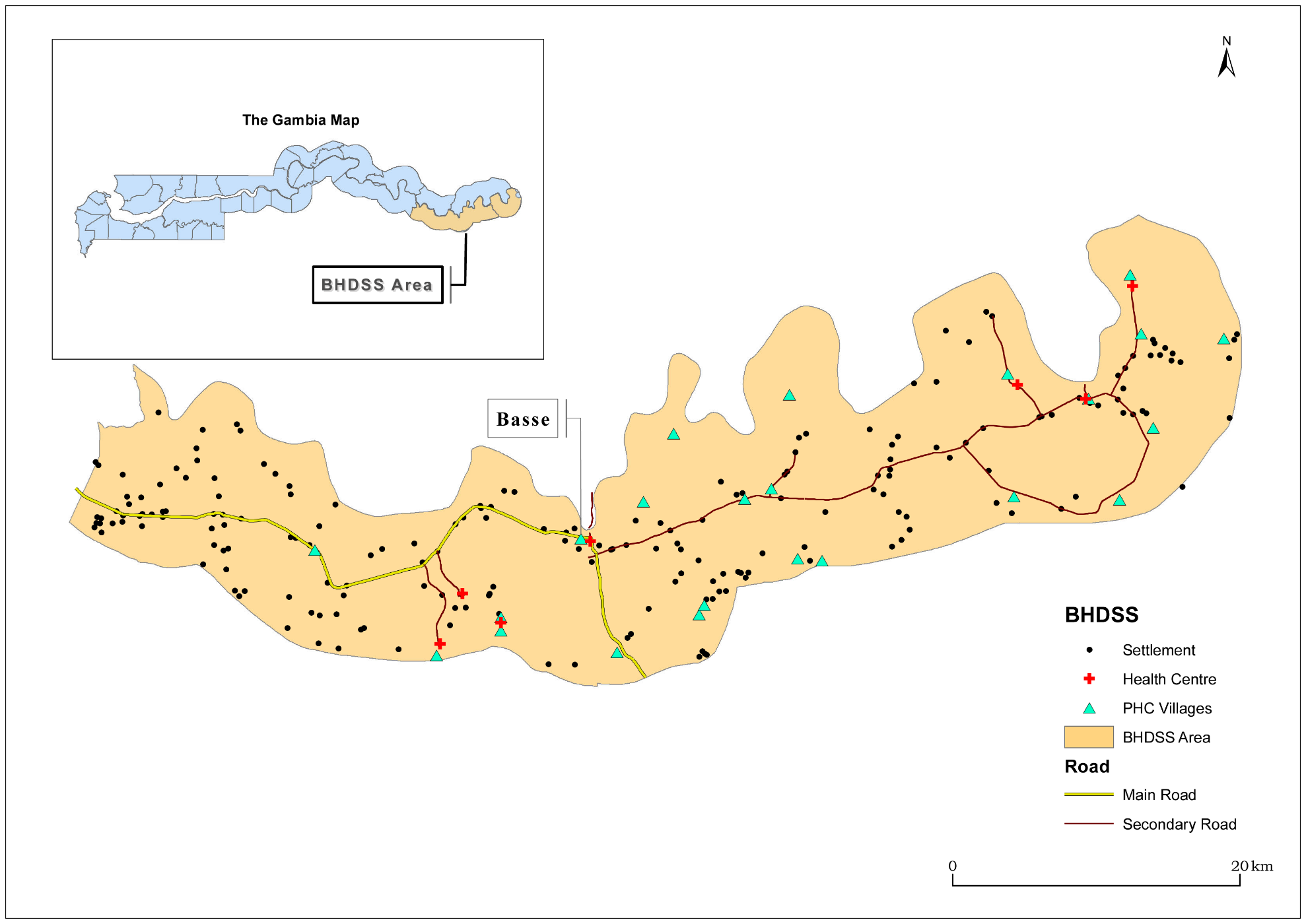


**The Gambia**
