# Supplementary material for: The Etiology of Childhood Pneumonia in The Gambia: Findings From the Pneumonia Etiology Research for Child Health (PERCH) Study
Source: Pediatr Infect Dis J. 2021 Aug 25;40(9):S7–S17. doi: 10.1097/INF.0000000000002766 (PMC8448408; doi:10.1097/INF.0000000000002766)
Supplement: Supplementary file 2 [file inf-40-s07-s002.docx]

**Supplemental Digital Content 2, Table:** **PERCH Algorithm for Specimen Collection and Laboratory Testing**

**A. Cases**

PERCH Algorithm for Specimen Collection and Laboratory Testing in Cases

| **Specimen** | **Subjects*** | **Assay** |
| --- | --- | --- |
| Acute blood | All (>95%) | Blood culture |
|  |  | Pneumococcal antigen testing on blood culture alarm (+) culture (−) specimens |
|  |  | Complete blood count with differential |
|  |  | Pneumococcus PCR |
|  |  | HIV test |
|  |  | Malaria antigen testing or microscopy (selected sites) |
|  |  | Serologic testing |
|  |  | C-reactive protein, other biomarkers |
|  |  | Host genetic studies |
| Urine | All (>95%) | Storage for future antigen testing, biomarkers |
| NP flocked swab (VTM) | All (>95%) | PCR for respiratory pathogens |
| NP rayon swab (STGG) | All (>95%) | Bacterial culture and serotyping for pneumococcus |
| Throat rayon swab | All (>95%) | PCR for respiratory pathogens |
| Induced sputum | All, except when contraindicated (>90%) | Microscopy, bacterial culture and AST |
|  |  | *Mycobacterium tuberculosis* microscopy, culture |
|  |  | PCR for respiratory pathogens |
| Lung aspirate | Select cases (<10%) | Microscopy, bacterial culture and AST |
|  |  | *M. tuberculosis* microscopy, culture |
|  |  | PCR for respiratory pathogens |
| Gastric aspirate | Select cases (<5%) | *M. tuberculosis* microscopy, culture |
| Pleural fluid | Select cases (<5%) | Microscopy, bacterial culture and AST |
|  |  | Cell count, protein, glucose |
|  |  | *M. tuberculosis* microscopy, culture |
|  |  | Antigen detection (pneumococcus) |
|  |  | PCR for respiratory pathogens |

Abbreviations: AST, antibiotic susceptibility testing; HIV, human immunodeficiency virus; NP, nasopharyngeal; PCR, polymerase chain reaction; PERCH, Pneumonia Etiology Research for Child Health; STGG, skim milk-tryptone-glucose-glycerin; VTM, viral transport media.

*Shown with the proportion of cases expected to have a specimen available.

**B. Controls**

| **Specimen*** | **Assay** |
| --- | --- |
| Acute blood | Pneumococcus PCR |
|  | Malaria antigen testing or microscopy (selected sites) |
|  | Biomarkers |
|  | Storage for future serologic testing |
|  | Host genetic studies |
| NP flocked swab (VTM) | PCR for respiratory pathogens |
| NP rayon swab (STGG) | Bacterial culture and serotyping for pneumococcus |
| Throat rayon swab | PCR for respiratory pathogens |
| Urine | Storage for future antigen testing |

Abbreviations: NP, nasopharyngeal; PCR, polymerase chain reaction; PERCH, Pneumonia Etiology Research for Child Health; STGG, skim milk-tryptone-glucose-glycerin; VTM, viral transport media.

*Each specimen type will be collected from each control subject.

**C. Laboratory Testing Strategy for Cases and Controls in the PERCH Project**

| **Specimen Type** | **Testing Methods** | **Target Pathogens** |
| --- | --- | --- |
| Whole blood | Culture* | Bacteria |
|  | Uniplex PCR | *Streptococcus pneumoniae* |
| Serum | Antibiotic bioassay | N/A |
| Combined nasopharyngeal and oropharyngeal swabs | Multiplex PCR | Bacteria, viruses, and *Pneumocystis jirovecii*** |
| Nasopharyngeal swab | Culture | *S. pneumoniae* |
| Induced sputum* | Microscopy/culture, multiplex PCR, mycobacterial culture | Bacteria, mycobacteria, viruses, and *P. jirovecii* |
| Lung aspirate* | Microscopy/culture, multiplex PCR, mycobacterial culture | Bacteria, mycobacteria, viruses, and *P. jirovecii* |
| Pleural fluid* | Microscopy/culture, antigen detection, multiplex PCR, mycobacterial culture | Bacteria, mycobacteria, viruses, and *P. jirovecii* |
| Gastric aspirate* | Mycobacterial culture (if no induced sputum obtained) | Mycobacteria |
| Urine | Antibiotic bioassay | N/A |

Abbreviations: N/A, not applicable; PCR, polymerase chain reaction; PERCH, Pneumonia Etiology Research for Child Health study.

*Cases only.

** The Fast Track Diagnostics Respiratory Pathogens 33 (FTD Resp-33) multiplex PCR kit which was used in the PERCH study includes the following 33 viral, bacterial and fungal targets:

- influenza A, B and C
- parainfluenza viruses types 1, 2, 3 and 4
- coronaviruses NL63, 229E OC43 and HKU1
- human metapneumovirus A/B
- human rhinovirus
- respiratory syncytial virus A/B
- adenovirus
- enterovirus
- parechovirus
- bocavirus
- cytomegalovirus
- *Pneumocystis jirovecii*
- *Mycoplasma pneumoniae*
- *Chlamydophila pneumoniae*
- *Streptococcus pneumoniae*
- *Haemophilus influenzae* type b
- *Haemophilus influenzae* species
- *Staphylococcus aureus*
- *Moraxella catarrhalis*
- *Bordetella pertussis*
- *Klebsiella pneumoniae*
- Legionella species
- Salmonella species
