# Supplementary material for: The Etiology of Childhood Pneumonia in The Gambia: Findings From the Pneumonia Etiology Research for Child Health (PERCH) Study
Source: Pediatr Infect Dis J. 2021 Aug 25;40(9):S7–S17. doi: 10.1097/INF.0000000000002766 (PMC8448408; doi:10.1097/INF.0000000000002766)
Supplement: Supplementary file 3 [file inf-40-s07-s003.docx]

**Supplemental Digital Content 3**

For more information about the sensitivity priors in the PERCH Integrated Etiology Analysis refer to Appendix Section III B 6 in The PERCH Study Group. Causes of severe pneumonia requiring hospital admission in children without HIV infection from Africa and Asia: the PERCH multi-country case-control study. Lancet. 2019; 6736(19):1-23.

**Table: Integrated etiology analysis input values for sensitivity and specificity of laboratory test measures^a^**

| **Specimen/test** | **Pathogen** | **Sensitivity Prior^a^** | | **Specificity** |
| --- | --- | --- | --- | --- |
|  |  | **Base** | **Reduced^b^** |  |
| Blood cultures^c^ | *-Streptococcus pneumoniae*  *-Haemophilus influenzae* | 5-20% | 1-13% | 100% |
|  | *-Moraxella catarrhalis*  *-Staphylococcus aureus*  -Nonfermentative gram-negative rods  -Candida species  -Non-pneumococcal streptococci, including enterococci | 5-15% | 1-10% |  |
|  | Salmonella species  Enterobacteriaceae  *Neisseria meningitidis* | 10-50% | 1-34% |  |
| NP/OP PCR | *-Streptococcus pneumoniae*  *-Haemophilus influenzae* | 50-90% | 15-55% | 1-Control prevalence (ref SDC 6) |
|  | *-*Salmonella species  -Legionella species | 0.5-90% | 0.5-90% |  |
|  | -All other PCR targets | 50-90% | 50-90% |  |
| Whole blood PCR | *- Streptococcus pneumoniae* | 12-65% | 12-65% | 1-Control prevalence (ref SDC 6) |
| Induced sputum | -*Mycobacterium tuberculosis* | 10-30% | 10-30% | 100% |

Abbreviation: SDC, Supplemental Digital Content.

a. Background information supporting choice of sensitivity priors provided in the all-site PERCH paper (The PERCH Study Group, 2019).

b. For some measurement-pathogen combinations the sensitivity priors were adjusted (reduced) for prior antibiotic exposure and low blood volume (blood culture only). For the children missing blood culture volume or antibiotic use we assumed adequate blood volume and no prior antibiotic use given the data for the other children in Bangladesh.

c. Direct evidence of the diagnostic sensitivity for *Streptococcus pneumoniae* and *Haemophilus influenzae* from vaccine probe studies. For all other pathogens we set the base blood culture sensitivity prior to 5-15%, with the exception of Salmonella species, Enterobacteriaceae and *Neisseria meningitidis*, for which we selected wider priors (10-50%) to reflect their greater uncertainty.
