# Supplementary material for: The Etiology of Childhood Pneumonia in The Gambia: Findings From the Pneumonia Etiology Research for Child Health (PERCH) Study
Source: Pediatr Infect Dis J. 2021 Aug 25;40(9):S7–S17. doi: 10.1097/INF.0000000000002766 (PMC8448408; doi:10.1097/INF.0000000000002766)
Supplement: Supplementary file 4 [file inf-40-s07-s004.docx]

**Supplemental Digital Content 4, Table: Specimen Collection, HIV-uninfected Cases and Controls**

|  | **All Cases** | **CXR+ Cases** | **All Controls** |
| --- | --- | --- | --- |
| **All** | 631 | 286 | 654 |
| **NP/OP VTM swab** | 621 (99.4) | 281 (98.9) | 646 (98.8) |
| **NP STGG swab** | 621 (99.4) | 281 (98.9) | 644 (98.5) |
| **Blood Culture** | 615 (97.5) | 279 (97.6) | -- |
| **Plain/red top tube** | 565 (90.4) | 261 (91.9) | 624 (95.4) |
| **EDTA tube 1 (CBC)** | 603 (96.5) | 274 (96.5) | 625 (95.6) |
| **Serum** | 601 (96.2) | 272 (95.8) | -- |
| **Convalescent serum** | 541 (97.3) | 243 (98) | -- |
| **Induced Sputum** |  |  |  |
| **Total** | 589 (93.3) | 261 (91.3) | -- |
| **Severe cases^a^** | 519 (96.6) | 233 (94.3) | -- |
| **Very severe cases^b^** | 70 (74.5) | 28 (71.8) | -- |
| **Urine** | 600 (96.0) | 272 (95.8) | 487 (74.5) |
| **Convalescent urine** | 291 (46.6) | 128 (45.1) | -- |
| **Lung Aspirate** |  |  |  |
| **Total** | 22 (3.5) | 21 (7.3) | -- |
| **Severe cases^a^** | 16 (3.0) | 15 (6.1) | -- |
| **Very severe cases^b^** | 6 (6.4) | 6 (15.4) | -- |
| **Pleural fluid** |  |  |  |
| **Total** | 3 (0.5) | 3 (1.0) | -- |
| **Severe cases^a^** | 0 (0.0) | 0 (0.0) | -- |
| **Very severe cases^b^** | 3 (3.2) | 3 (7.7) | -- |

Abbreviations: EDTA, Ethylenediaminetetraacetic acid; STGG, skim milk-tryptone-glucose-glycerin; VTM, viral transport media.

^a^Proportion among severe cases.

^b^Proportion among very severe cases.

Lung aspirates and pleural fluid represent all specimens collected; specimens collected more than 3 days after enrollment were excluded from analyses.
