# Supplementary material for: The Etiology of Childhood Pneumonia in The Gambia: Findings From the Pneumonia Etiology Research for Child Health (PERCH) Study
Source: Pediatr Infect Dis J. 2021 Aug 25;40(9):S7–S17. doi: 10.1097/INF.0000000000002766 (PMC8448408; doi:10.1097/INF.0000000000002766)
Supplement: Supplementary file 5 [file inf-40-s07-s005.docx]

**Supplemental Digital Content 5, Table: List of children positive for *S. pneumoniae* by Blood Culture or Lung Aspirate**

**A. *S. pneumoniae***

| **Number** | **Age (m)** | **Blood culture results** | **Lung aspirate culture results** | **Lung aspirate PCR results** | **S. pneu serotype detected on invasive isolate** | **CXR finding** | **Pneumonia severity** | **PCV doses received** |
| --- | --- | --- | --- | --- | --- | --- | --- | --- |
| 1 | 1 | S. pneu | ND | ND | 2 (NVT) | Uninterpretable | Very Severe | 0 |
| 2 | 3 | S. pneu | ND | ND | 12F (NVT) | Consolidation | Very Severe | 1 |
| 3 | 3 | S. pneu | ND | ND | 2 (NVT) | Normal | Very Severe | 1 |
| 4 | 5 | S. pneu | ND | ND | 12F (NVT) | Other Infiltrate | Severe | 2 |
| 5 | 6 | S. pneu | S. pneu | Missing | BC and LA: 12F (NVT) | Consolidation | Severe | 3 |
| 6 | 7 | S. pneu | ND | ND | 46 (NVT) | Normal | Very Severe | Missing |
| 7 | 9 | Negative | S. pneu  H. inf | S. pneu  H. inf  M. cat | 20 (NVT) | Consolidation | Very Severe | 3 |
| 8 | 10 | Negative | Negative | S. pneu | NA | Consolidation | Severe | 3 |
| 9 | 13 | S. pneu | ND | ND | 35B (NVT) | Consolidation | Very Severe | 3 |
| 10 | 14 | S. pneu | ND | ND | 12F (NVT) | Consolidation | Very Severe | 3 |
| 11 | 15 | S. pneu | ND | ND | **19F (VT)** | Other Infiltrate | Very Severe | 3 |
| 12 | 16 | Negative | S. pneu | Missing | **6A (VT)** | Consolidation | Severe | 3 |
| 13 | 22 | Negative | S. pneu | S. pneu  M. cat | **1 (VT)** | Consolidation | Very Severe | 3 |
| 14 | 23 | Negative | S. pneu | S. pneu  M. cat | **5 (VT)** | Consolidation | Severe | 3 |
| 15 | 23 | S. pneu | Negative | Negative | **1 (VT)** | Consolidation with Other Infiltrate | Very Severe | 3 |
| 16 | 41 | Negative | Negative | S. pneu | **1 (VT)^a^** | Consolidation | Very Severe | 3 |

Abbreviations: S. pneu, *Streptococcus pneumoniae*; H. inf, *Haemophilus influenzae*; M. cat, *Moraxella catarrhalis*; ND, not done; NA, not available (positive by PCR only), PCV, pneumococcal conjugate vaccine; BC, blood culture; LA, lung aspirate. Bold = PCV13 serotype detected on invasive isolate. Lung aspirate results restricted to specimens obtained within 3 days of enrollment and those pathogens determined by the clinical review team to be non-contaminants.

a. Positive by lung aspirate PCR so no serotype available from an invasive isolate. However, the serotype detected on induced sputum (ST=1) was assumed to the ST that would have been detected on lung aspirate since it is rarely detected in healthy children.

**Supplemental Digital Content 5, Table: List of children positive for *H. influenzae* by Blood Culture or Lung Aspirate**

**B. *H. influenzae***

| **Number** | **Age (m)** | **Blood culture results** | **Lung aspirate culture results** | **Lung aspirate PCR results** | **H. inf serotype detected on invasive isolate** | **CXR finding** | **Pneumonia severity** | **Penta doses received** |
| --- | --- | --- | --- | --- | --- | --- | --- | --- |
| 1 | 3 | H. inf | ND | ND | b | Normal | Very Severe | 1 |
| 2 | 5 | H. inf | Negative | H. inf | BC: a | Consolidation | Severe | 1 |
| 3 | 9 | Negative | S. pneu  H. inf | S. pneu  H. inf  M. cat | Non-b^a^ | Consolidation | Very Severe | 3 |
| 4 | 13 | H. inf | ND | ND | NT | Other Infiltrate | Severe | 3 |

Abbreviations: BC, blood culture; LA, lung aspirate; S. pneu, *Streptococcus pneumoniae*; H. inf, *Haemophilus influenzae*; M. cat, *Moraxella catarrhalis*; HMPV, Human metapneumovirus A/B; ND, not done; NT, non-typeable. Lung aspirate results restricted to specimens obtained within 3 days of enrollment and those pathogens determined by the clinical review team to be non-contaminants.

a. Case was positive for *H. influenzae* by lung aspirate culture and PCR; serotyping results for culture isolate was missing but was *H. influenzae* non-b by PCR.
