# Supplementary material for: The Etiology of Childhood Pneumonia in The Gambia: Findings From the Pneumonia Etiology Research for Child Health (PERCH) Study
Source: Pediatr Infect Dis J. 2021 Aug 25;40(9):S7–S17. doi: 10.1097/INF.0000000000002766 (PMC8448408; doi:10.1097/INF.0000000000002766)
Supplement: Supplementary file 6 [file inf-40-s07-s006.docx]

**Supplemental Digital Content 6, Table: Detection of Organisms by Nasopharyngeal-Oropharyngeal and Whole Blood Specimens from Cases and Controls, by CXR status**

|  | | | | **Odds Ratio (95% CI)^a^** | | |
| --- | --- | --- | --- | --- | --- | --- |
|  | **All Cases**  **(N=609)** | **CXR+ Cases (N=273)** | **All Controls (N=624)** | **All Cases vs. All Controls** | **CXR+ Cases vs. All Controls** |  |
| **NP/OP PCR** | | | | | | |
| **Pathogen** | | | | | | |
| **Any pathogen** | 609 (100) | 273 (100) | 621 (99.5) |  |  |  |
| **Any pathogen, with thresholds applied^b^** | 601 (98.7) | 269 (98.5) | 607 (97.3) |  |  |  |
| **Bacteria** | | | | | | |
| **Any bacteria** | 585 (96.1) | 263 (96.3) | 620 (99.4) |  |  |  |
| **Any bacteria, with thresholds applied for *S. pneumoniae, H. influenzae* and *P. jirovecii*** | 559 (91.8) | 252 (92.3) | 602 (96.5) |  |  |  |
| ***S. pneumoniae*** | 519 (85.2) | 233 (85.3) | 553 (88.6) | 0.91 (0.60, 1.37) | 1.09 (0.64, 1.88) |  |
| **>6.9 log_10_ copies/ml** | 104 (17.1) | 55 (20.1) | 51 (8.2) | **2.31 (1.52, 3.52)** | **3.02 (1.81, 5.05)** |  |
| **Among those with >6.9 log10 copies/ml** |  |  |  |  |  |  |
| **PCV13-type** | 31 (29.8) | 17 (30.9) | 16 (31.4) | 1.95 (0.95, 3.97) | **2.63 (1.12, 6.18)** |  |
| **Non PCV13-type** | 73 (70.2) | 36 (65.5) | 35 (68.6) | **2.21 (1.37, 3.58)** | **2.65 (1.48, 4.77)** |  |
| ***H. influenzae*** | 454 (74.6) | 207 (75.8) | 454 (72.8) | 0.92 (0.68, 1.25) | 1.02 (0.68, 1.54) |  |
| ***H. influenzae* not type b** | 432 (70.9) | 195 (71.4) | 427 (68.4) | 0.93 (0.69, 1.26) | 1.02 (0.67, 1.54) |  |
| ***H. influenzae* not type b > 5.9 log_10_ copies/ml** | 278 (45.6) | 124 (45.4) | 246 (39.4) | 1.02 (0.77, 1.34) | 0.99 (0.68, 1.42) |  |
| ***H. influenzae* type b** | 22 (3.6) | 12 (4.4) | 27 (4.3) | 0.76 (0.38, 1.51) | 1.07 (0.45, 2.55) |  |
| ***H. influenzae* type b > 5.9 log_10_ copies/ml** | 10 (1.6) | 6 (2.2) | 3 (0.5) | 3.05 (0.71, 13.10) | 3.40 (0.63, 18.18) |  |
| ***S. aureus*** | 40 (6.6) | 15 (5.5) | 46 (7.4) | 0.76 (0.46, 1.27) | 0.53 (0.25, 1.11) |  |
| ***C. pneumoniae*** | 5 (0.8) | 3 (1.1) | 11 (1.8) | 0.53 (0.16, 1.72) | 0.73 (0.17, 3.16) |  |
| ***M. catarrhalis*** | 527 (86.5) | 236 (86.4) | 586 (93.9) | **0.33 (0.21, 0.54)** | **0.30 (0.16, 0.56)** |  |
| ***M. pneumoniae*** | 9 (1.5) | 6 (2.2) | 7 (1.1) | 1.12 (0.28, 4.49) | 1.24 (0.24, 6.36) |  |
| **Salmonella species** | 12 (2.0) | 9 (3.3) | 13 (2.1) | 0.69 (0.24, 1.94) | 1.12 (0.33, 3.75) |  |
| **Legionella** | 0 (0.0) | 0 (0.0) | 0 (0.0) | -- | -- |  |
| ***B. pertussis*** | 1 (0.2) | 1 (0.4) | 3 (0.5) | 0.40 (0.03, 5.69) | 0.84 (0.06, 11.69) |  |
| **Fungi** |  |  |  |  |  |  |
| ***P. jirovecii*** | 31 (5.1) | 14 (5.1) | 51 (8.2) | **0.45 (0.25, 0.80)** | **0.33 (0.14, 0.78)** |  |
| **> 4 log_10_ copies/ml** | 13 (2.1) | 7 (2.6) | 22 (3.5) | 0.43 (0.18, 1.05) | 0.38 (0.12, 1.26) |  |
| **Virus** | | | | | | |
| **Any virus** | 567 (93.1) | 256 (93.8) | 498 (79.8) |  |  |  |
| **Any virus, with thresholds applied for CMV** | 540 (88.7) | 246 (90.1) | 440 (70.5) |  |  |  |
| **Adenovirus** | 60 (9.9) | 27 (9.9) | 74 (11.9) | 1.08 (0.72, 1.63) | 1.11 (0.64, 1.93) |  |
| **CMV** | 321 (52.7) | 156 (57.1) | 331 (53.0) | 1.09 (0.84, 1.42) | **1.50 (1.05, 2.14)** |  |
| **> 4.9 log_10_ copies/ml** | 133 (21.8) | 60 (22.0) | 138 (22.1) | 0.72 (0.52, 1.01) | 0.78 (0.50, 1.21) |  |
| **Coronavirus 43** | 22 (3.6) | 9 (3.3) | 27 (4.3) | 1.16 (0.61, 2.22) | 1.41 (0.59, 3.36) |  |
| **Coronavirus 63** | 17 (2.8) | 7 (2.6) | 20 (3.2) | 0.92 (0.42, 1.99) | 0.80 (0.27, 2.34) |  |
| **Coronavirus HKU** | 9 (1.5) | 6 (2.2) | 14 (2.2) | 0.84 (0.33, 2.14) | 1.49 (0.52, 4.29) |  |
| **Coronavirus 229** | 13 (2.1) | 6 (2.2) | 14 (2.2) | 0.86 (0.34, 2.19) | 0.74 (0.22, 2.45) |  |
| **HBOV** | 98 (16.1) | 45 (16.5) | 99 (15.9) | 1.28 (0.90, 1.82) | 1.27 (0.80, 2.02) |  |
| **HMPV A/B** | 40 (6.6) | 19 (7.0) | 13 (2.1) | **5.12 (2.63, 9.97)** | **6.12 (2.82, 13.29)** |  |
| **Influenza A** | 23 (3.8) | 14 (5.1) | 7 (1.1) | **3.77 (1.47, 9.63)** | **6.01 (2.15, 16.81)** |  |
| **Influenza B** | 4 (0.7) | 2 (0.7) | 7 (1.1) | 0.64 (0.16, 2.60) | 0.77 (0.13, 4.45) |  |
| **Influenza C** | 7 (1.1) | 3 (1.1) | 7 (1.1) | 1.06 (0.31, 3.61) | 0.80 (0.14, 4.53) |  |
| **Measles^c^** | 0 (0.0) | 0 (0.0) | -- | -- | -- |  |
| **Parainfluenza 1** | 27 (4.4) | 14 (5.1) | 6 (1.0) | **7.88 (3.10, 20.03)** | **10.49 (3.71, 29.64)** |  |
| **Parainfluenza 2** | 21 (3.4) | 8 (2.9) | 13 (2.1) | 1.37 (0.45, 4.13) | 0.94 (0.21, 4.16) |  |
| **Parainfluenza 3** | 53 (8.7) | 17 (6.2) | 18 (2.9) | **4.34 (2.41, 7.82)** | **3.24 (1.52, 6.90)** |  |
| **Parainfluenza 4** | 22 (3.6) | 9 (3.3) | 14 (2.2) | 1.41 (0.49, 4.03) | 1.33 (0.34, 5.15) |  |
| **PV/EV** | 67 (11.0) | 27 (9.9) | 64 (10.3) | 1.31 (0.86, 2.01) | 1.00 (0.56, 1.78) |  |
| **Rhinovirus** | 129 (21.2) | 52 (19.0) | 162 (26.0) | 1.18 (0.86, 1.61) | 0.94 (0.61, 1.43) |  |
| **RSV** | 197 (32.3) | 101 (37.0) | 29 (4.6) | **11.93 (7.71, 18.46)** | **14.59 (8.94, 23.82)** |  |
| **Whole Blood PCR** | | | | | | |
| ***S. pneumoniae*** | 60 (9.5) | 25 (8.7) | 47 (7.2) | 1.28 (0.86, 1.92) | 1.16 (0.69, 1.93) |  |
| **>2.2 log_10_ copies/ml** | 38 (6.0) | 16 (5.6) | 29 (4.4) | 1.32 (0.80, 2.18) | 1.20 (0.64, 2.26) |  |

Abbreviations: HBOV, Human bocavirus; CMV, cytomegalovirus; CXR, chest x-ray; HMPV, Human metapneumovirus A/B; NP/OP, nasopharyngeal/oropharyngeal; PCV, pneumococcal conjugate vaccine; PV/EV, Parechovirus/Enterovirus; RSV, Respiratory syncytial virus A/B.

a. NP/OP PCR odds ratios adjusted for age (months) and all other pathogens detected on NP/OP PCR. Whole blood PCR odds ratios adjusted for age in months.

b. Threshold defined using NP/OP PCR density for 4 pathogens: *P. jirovecii*, 4 log10 copies/mL; *H. influenzae*, 5.9 log10 copies/mL; CMV, 4.9 log10 copies/mL; *S. pneumoniae*, 6.9 log10 copies/mL.

c. Five cases, including 3 CXR+ cases, had clinical signs or history or measles and were tested for measles by NP/OP PCR.
