# Supplementary material for: The Etiology of Childhood Pneumonia in The Gambia: Findings From the Pneumonia Etiology Research for Child Health (PERCH) Study
Source: Pediatr Infect Dis J. 2021 Aug 25;40(9):S7–S17. doi: 10.1097/INF.0000000000002766 (PMC8448408; doi:10.1097/INF.0000000000002766)
Supplement: Supplementary file 7 [file inf-40-s07-s007.docx]

**Supplemental Digital Content 7, Table: Codetection of Organisms in Nasopharyngeal-Oropharyngeal Specimens Collected from Cases and Controls, by CXR status**

|  | **All Cases N=609** | **CXR+ Cases N=273** | **All Controls N=624** | **All Cases vs All Controls**  **p-value** | **CXR^+^ Cases vs All Controls**  **p-value** |
| --- | --- | --- | --- | --- | --- |
| Mean (SD) number of organisms, any positivity | 4.48 (1.38) | 4.56 (1.39) | 4.24 (1.42) |  |  |
| Median (IQR) | 4.0 (4.0, 5.0) | 5.0 (4.0, 5.0) | 4.0 (3.0, 5.0) |  |  |
| 0 | 0 (0) | 0 (0) | 3 (0.5) | 0.0518 | 0.1134 |
| 1 | 17 (2.8) | 8 (2.9) | 16 (2.6) |  |  |
| 2 | 32 (5.3) | 11 (4.0) | 46 (7.4) |  |  |
| 3 | 72 (11.8) | 33 (12.1) | 112 (17.9) |  |  |
| 4 | 488 (80.1) | 221 (81.0) | 447 (71.6) |  |  |
| Mean (SD) number of organisms, above threshold^a^ | 3.19 (1.38) | 3.25 (1.42) | 2.75 (1.42) |  |  |
| Median (IQR) | 3.0 (2.0, 4.0) | 3.0 (2.0, 4.0) | 3.0 (2.0, 4.0) |  |  |
| 0 | 8 (1.3) | 4 (1.5) | 17 (2.7) | <.0001 | 0.0012 |
| 1 | 59 (9.7) | 23 (8.4) | 99 (15.9) |  |  |
| 2 | 122 (20.0) | 57 (20.9) | 185 (29.6) |  |  |
| 3 | 181 (29.7) | 78 (28.6) | 148 (23.7) |  |  |
| 4 | 239 (39.2) | 111 (40.7) | 175 (28.0) |  |  |
| Pathogen patterns, any positivity |  |  |  |  |  |
| Single bacteria | 4 (0.7) | 2 (0.7) | 15 (2.4) | <.0001 | <.0001 |
| 2 or more bacteria | 38 (6.2) | 15 (5.5) | 108 (17.3) |  |  |
| Single virus | 13 (2.1) | 6 (2.2) | 1 (0.2) |  |  |
| 2 or more viruses | 11 (1.8) | 4 (1.5) | 0 (0) |  |  |
| Bacterial-Viral | 543 (89.2) | 246 (90.1) | 497 (79.6) |  |  |
| Pathogen patterns, above threshold^a^ |  |  |  |  |  |
| Single bacteria | 37 (6.1) | 13 (4.8) | 96 (15.4) | <.0001 | <.0001 |
| 2 or more bacteria | 24 (3.9) | 10 (3.7) | 71 (11.4) |  |  |
| Single virus | 23 (3.8) | 11 (4.0) | 3 (0.5) |  |  |
| 2 or more viruses | 19 (3.1) | 6 (2.2) | 2 (0.3) |  |  |
| Bacterial-Viral | 498 (81.8) | 229 (83.9) | 435 (69.7) |  |  |

Analysis population was restricted to HIV-uninfected cases and controls with available NPPCR data. P-values are from logistic regression adjusted for age in months

a. Threshold defined using NP/OP PCR density for 4 pathogens: *P. jirovecii*, 4 log10 copies/mL; *H. influenzae*, 5.9 log10 copies/mL; CMV, 4.9 log10 copies/mL; *S. pneumoniae*, 6.9 log10 copies/mL).
