# Supplementary material for: The Etiology of Childhood Pneumonia in The Gambia: Findings From the Pneumonia Etiology Research for Child Health (PERCH) Study
Source: Pediatr Infect Dis J. 2021 Aug 25;40(9):S7–S17. doi: 10.1097/INF.0000000000002766 (PMC8448408; doi:10.1097/INF.0000000000002766)
Supplement: Supplementary file 8 [file inf-40-s07-s008.docx]

**Supplemental Digital Content 8, Figure. NP/OP Prevalence in CXR+ Cases and Controls and Odds Ratios**


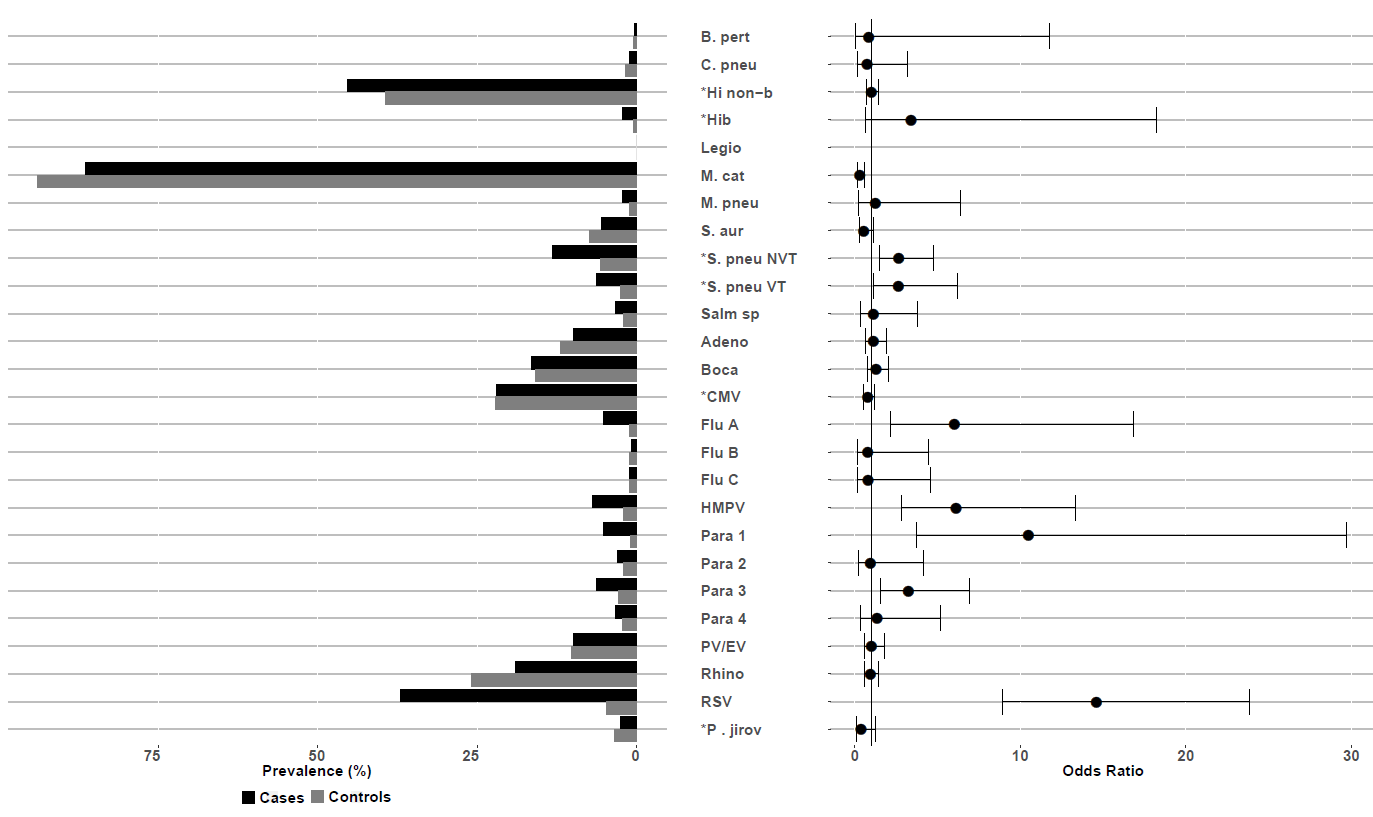


Abbreviations: NP/OP, nasopharyngeal/oropharyngeal; CXR+, chest radiograph positive (consolidation and/or other infiltrate); VT, PCV13 vaccine type; NVT, non PCV13 vaccine type; Adeno, Adenovirus; B. pert, *Bordetella pertussis*; Boca, Human bocavirus; C. pneu, *Chlamydophila pneumoniae*; CMV, cytomegalovirus; Flu, influenza virus; HCoV, Human coronavirus; Hib, *Haemophilus influenzae* type b; Hi non-b, *Haemophilus influenzae* non-b; HMPV, Human metapneumovirus A/B; Legio, Legionella; M. cat, *Moraxella catarrhalis*; M. pneu, *Mycoplasma pneumoniae*; Para, Parainfluenza virus; P. jirov, *Pneumocystis jirovecii*; PV/EV, Parechovirus/Enterovirus; Rhino, Rhinovirus; RSV, Respiratory syncytial virus A/B; S. aur, *Staphylococcus aureus*; S. pneu, *Streptococcus pneumoniae*; Salm sp, Salmonella species.

Pathogens are ordered alphabetically among bacteria and then viruses and fungi.

*Prevalence defined using NP/OP PCR density thresholds for 4 pathogens: *P. jirovecii*, 4 log_10_ copies/mL; *H. influenzae*, 5.9 log_10_ copies/mL; CMV, 4.9 log_10_ copies/mL; *S. pneumoniae*, 6.9 log_10_ copies/mL). Refer to Supplementary Table 6 for NP/OP PCR results based on positivity.

Odds ratios adjusted for age (months) and presence of other pathogens detected by NP/OP PCR.
