# Supplementary material for: The Etiology of Childhood Pneumonia in The Gambia: Findings From the Pneumonia Etiology Research for Child Health (PERCH) Study
Source: Pediatr Infect Dis J. 2021 Aug 25;40(9):S7–S17. doi: 10.1097/INF.0000000000002766 (PMC8448408; doi:10.1097/INF.0000000000002766)
Supplement: Supplementary file 9 [file inf-40-s07-s009.docx]

**Supplemental Digital Content 9, Table.** **Integrated etiology results for HIV-uninfected Cases**

| **Aetiology** | **All Cases (N=631)** | **CXR+ Cases (N=286)** |
| --- | --- | --- |
|  | **Etiologic Fraction (95% CI)** | **Etiologic Fraction (95% CI)** |
| **Bacteria** |  |  |
| *B. pertussis* | 0.0 (0.0, 0.3) | 0.1 (0.0, 0.7) |
| *C. pneumoniae* | 0.1 (0.0, 0.5) | 0.1 (0.0, 0.7) |
| Enterobacteriaceae | 2.8 (1.0, 6.5) | 2.0 (0.4, 6.7) |
| *H. influenzae* | 3.8 (1.1, 8.0) | 4.7 (1.4, 9.9) |
| Type b | 0.3 (0.2, 1.0) | 0.2 (0.0, 1.1) |
| Non-b | 3.4 (0.8, 7.6) | 4.6 (1.4, 9.9) |
| Legionella species | 0.2 (0.0, 1.5) | 0.4 (0.0, 2.8) |
| *M. catarrhalis* | 3.8 (0.6, 9.1) | 3.2 (0.7, 7.7) |
| *M. pneumoniae* | 0.1 (0.0, 0.5) | 0.2 (0.0, 1.1) |
| *M. tuberculosis* | 5.1 (2.4, 9.4) | 8.3 (4.2, 14.1) |
| *N. meningitidis* | 1.4 (0.3, 3.7) | 1.1 (0.4, 3.2) |
| Non-fermenting gram-negative rods | 1.7 (0.2, 5.4) | 0.6 (0.0, 3.9) |
| Other streptococci and enterococci | 0.4 (0.0, 2.3) | 0.6 (0.0, 3.5) |
| *S. aureus* | 2.0 (0.6, 3.9) | 1.5 (0.7, 3.2) |
| *S. pneumoniae* | 9.0 (6.0, 12.8) | 13.0 (9.2, 17.6) |
| VT | 3.3 (1.6, 5.7) | 5.5 (3.2, 8.5) |
| NVT | 5.8 (3.4, 8.9) | 7.5 (4.2, 11.6) |
| Salmonella species | 1.1 (0.3, 2.9) | 0.9 (0.4, 2.5) |
| **Fungi** |  |  |
| *Candida* species | 1.7 (0.2, 5.0) | 4.0 (0.4, 10.6) |
| *P. jirovecii* | 0.1 (0.0, 0.6) | 0.2 (0.0, 1.1) |
| **Viruses** |  |  |
| Adenovirus | 0.7 (0.0, 2.9) | 0.7 (0.0, 3.2) |
| Bocavirus | 0.9 (0.0, 3.7) | 0.8 (0.0, 3.9) |
| CMV | 0.2 (0.0, 1.3) | 0.5 (0.0, 2.5) |
| Coronavirus | 0.2 (0.0, 1.1) | 0.3 (0.0, 1.4) |
| HMPV A/B | 5.4 (3.1, 8.1) | 4.6 (1.8, 7.7) |
| Influenza | 1.2 (0.2, 2.9) | 2.2 (0.0, 4.9) |
| A | 0.9 (0.0, 2.3) | 1.8 (0.0, 4.2) |
| B | 0.2 (0.0, 1.0) | 0.3 (0.0, 1.8) |
| C | 0.2 (0.0, 1.0) | 0.1 (0.0, 1.1) |
| Parainfluenza | 13.9 (10.2, 18.0) | 9.2 (4.6, 13.7) |
| 1 | 4.5 (2.8, 6.7) | 4.5 (2.1, 7.0) |
| 2 | 0.5 (0.0, 1.9) | 0.5 (0.0, 2.1) |
| 3 | 7.3 (4.7, 10.6) | 3.7 (0.4, 7.0) |
| 4 | 1.6 (0.0, 3.6) | 0.6 (0.0, 2.5) |
| PV/EV | 2.4 (0.0, 5.4) | 0.8 (0.0, 3.2) |
| Rhinovirus | 5.2 (0.0, 9.4) | 1.7 (0.0, 6.7) |
| RSV A/B | 35.9 (31.0, 41.7) | 37.3 (32.0, 43.7) |
| Not otherwise specified | 0.8 (0.0, 4.5) | 1.1 (0.0, 6.7) |
| **Summary Estimates** |  |  |
| Bacteria** | 26.2 (19.3, 34.1) | 28.0 (21.1, 35.9) |
| Viruses | 66.0 (57.5, 73.4) | 58.0 (49.6, 66.2) |

Abbreviation: CI, credible interval, VT, vaccine-type, PV/EV, parechovirus/enterovirus; CMV, cytomegalovirus; RSV, respiratory syncytial virus.

**The bacteria summary estimate excludes *M. tuberculosis*.

CXR+ defined as consolidation and/or other infiltrate on chest radiograph.

For pathogens presented grouped in Figure 3a (e.g., Parainfluenza virus types 1, 2, 3 and 4), both grouped and subspecies level results are presented here, with subspecies level results in gray.

Not Otherwise Specified represents pathogens not tested for. Other Strep includes *Streptococcus pyogenes* and *Enterococcus faecium*. Nonfermentative gram-negative rods includes Acinetobacter species and Pseudomonas species. Enterobacteriaceae includes *E. coli*, Enterobacter species, and Klebsiella species, excluding mixed gram-negative rods.
