# Supplementary material for: The Etiology of Childhood Pneumonia in The Gambia: Findings From the Pneumonia Etiology Research for Child Health (PERCH) Study
Source: Pediatr Infect Dis J. 2021 Aug 25;40(9):S7–S17. doi: 10.1097/INF.0000000000002766 (PMC8448408; doi:10.1097/INF.0000000000002766)
Supplement: Supplementary file 11 [file inf-40-s07-s011.docx]

**Supplemental Digital Content 11, Figure. Integrated Etiology Results, CXR+ HIV- Cases, < 1 vs > 1 year of age**


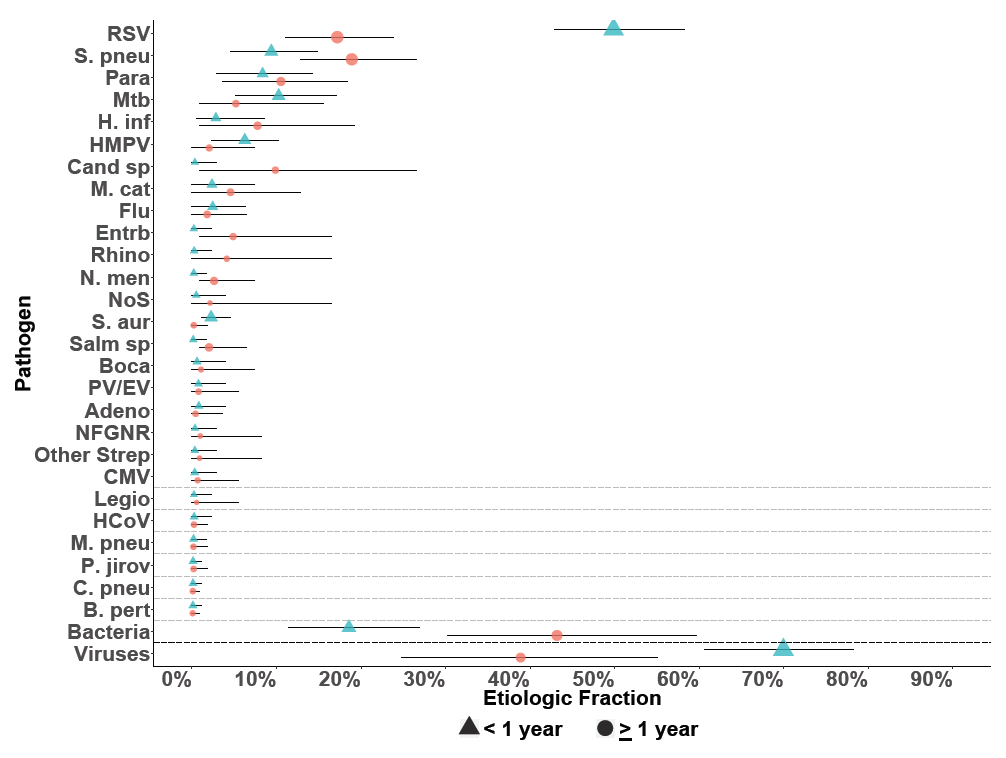


Abbreviations: Adeno, Adenovirus; B. pert, *Bordetella pertussis*; Boca, Human bocavirus; C. pneu, *Chlamydophila pneumoniae;* Cand sp, Candida species; CMV, cytomegalovirus; Entrb, Enterobacteriaceae; Flu, influenza virus A, B and C; H. inf, *Haemophilus influenzae*; HCoV, Coronavirus; HMPV, Human metapneumovirus A/B; Legio, Legionella species; M. cat, *Moraxella catarrhalis*; M. pneu, *Mycoplasma pneumoniae*; Mtb, *Mycobacterium tuberculosis*; NFGNR, Nonfermentative gram-negative rods; N. men, *Neiserria meningitidis*; NoS, Not Otherwise Specified (i.e., pathogens not tested for); P. jirov, *P. jirovecii*; Para, Parainfluenza virus types 1, 2, 3 and 4; PV/EV, Parechovirus/Enterovirus; Rhino, Human rhinovirus; RSV, Respiratory syncytial virus A/B; S. aur, *Staphylococcus aureus*; S. pneu, *Streptococcus pneumoniae*; Salm sp, Salmonella species.

Other Strep includes *Streptococcus pyogenes* and *Enterococcus faecium*. NFGNR includes Acinetobacter species and Pseudomonas species. Enterobacteriaceae includes *E. coli*, Enterobacter species, and Klebsiella species, excluding mixed gram-negative rods.

CXR+ defined as consolidation and/or other infiltrate on chest radiograph. Bacterial summary excludes Mtb.

Pathogens estimated at the subspecies level but grouped to the species level for display (Parainfluenza virus type 1, 2, 3 and 4; *S. pneumoniae* PCV 13 and *S. pneumoniae* non PCV 13 types; *H. influenzae* type b and *H. influenzae* non b; influenza A, B, and C).

Description of symbols: Line represents the 95% credible interval. The size of the symbol is scaled based on the ratio of the estimated etiologic fraction to its standard error. Of two identical aetiologic fraction estimates, the estimate associated with a larger symbol is more informed by the data than the priors.

Sample sizes: CXR+ < 1 year, N=177; CXR+ > 1 year, N=109.
