# Supplementary material for: The Etiology of Childhood Pneumonia in The Gambia: Findings From the Pneumonia Etiology Research for Child Health (PERCH) Study
Source: Pediatr Infect Dis J. 2021 Aug 25;40(9):S7–S17. doi: 10.1097/INF.0000000000002766 (PMC8448408; doi:10.1097/INF.0000000000002766)
Supplement: Supplementary file 14 [file inf-40-s07-s014.docx]

**Supplemental Digital Content 14, Table: Laboratory findings from cases who died in hospital, HIV-uninfected Cases**

| **Case** | **Severity** | **Age (m)** | **Chest**  **x-ray** | **Weight for height** | **Pathogens Detected** | | | | |
| --- | --- | --- | --- | --- | --- | --- | --- | --- | --- |
|  |  |  |  |  | **Blood culture** | **NP/OP PCR** | **TB positive on induced sputum** | **Lung aspirate** | **Pleural fluid** |
| 1 | Very Severe | 6 | PEP | Normal  (>-2 SDs and <+2 SDs) | Neg | CMV^a^  HCoV 43  C. pneu  H. inf^a^  M. cat  S. pneu^a^ | ND | ND | S. aur |
| 2 | Very Severe | 10 | PEP | Outlier low  (z-score <-5 SDs) | Neg | CMV  H. inf  M. cat  S. pneu PV/EV | Pos | ND | ND |
| 3 | Very Severe | 2 | Normal | Moderate  (>-3 SDs and  <-2 SDs) | Neg | CMV  Boca  H. inf  P. jirov  S. pneu | Neg | ND | ND |
| 4 | Very Severe | 41 | Normal | Normal  (>-2 SDs and <+2 SDs) | N. men | CMV  H. inf^a^  M. cat  S. pneu^a^ | ND | ND | ND |
| 5 | Very Severe | 16 | OI | Moderate  (>-3 SDs and  <-2 SDs) | E. col | HCoV 229 HCoV HKU  H. inf  S. aur | Neg | ND | ND |
| 6 | Very Severe | 15 | OI | Normal  (>-2 SDs and <+2 SDs) | S. pneu | CMV  H. inf^a^ HMPV A/B M. cat S.pneu^a^  S. aur | ND | ND | ND |
| 7 | Very Severe | 3 | Normal | Normal  (>-2 SDs and <+2 SDs) | S. pneu | Adeno  H. inf^a^  M. cat  Para 3  P. jirov^a^  S. pneu^a^ Rhino | ND | ND | ND |
| 8 | Very Severe | 16 | OI | Severe  (>-5 SDs and  <-3 SDs) | Neg | CMV^a^  H. inf^a^  M. cat  S. pneu^a^ Rhino  S. aur | ND | ND | ND |
| 9 | Very Severe | 14 | PEP | Normal  (>-2 SDs and <+2 SDs) | S. pneu | CMV  Boca  H. inf^a^  M. cat  Para 3  S. pneu^a^ | ND | ND | ND |
| 10 | Very Severe | 5 | OI | Normal  (>-2 SDs and <+2 SDs) | Neg | CMV^a^  H. inf  M. cat  S. pneu | Neg | ND | ND |
| 11 | Very Severe | 14 | Normal | Severe  (>-5 SDs and  <-3 SDs) | Neg | CMV  M. cat  Rhino  S. aur | ND | ND | ND |
| 12 | Severe | 11 | Both PEP/OI | Severe  (>-5 SDs and  <-3 SDs) | Neg | ND | ND | ND | ND |
| 13 | Severe | 5 | PEP | Moderate  (>-3 SDs and  <-2 SDs) | Neg | Adeno  Boca  H. inf^a^  M. cat  S. pneu^a^ Rhino | Neg | ND | ND |
| 14 | Severe | 7 | Normal | Moderate  (>-3 SDs and  <-2 SDs) | Neg | H. inf^a^  Para 1 | Neg | ND | ND |
| 15 | Very Severe | 6 | PEP | Moderate  (>-3 SDs and  <-2 SDs) | Neg | H. inf  CMV^a^  M. cat  S. pneu | Neg | ND | ND |
| 16 | Very Severe | 17 | ND | Moderate  (>-3 SDs and  <-2 SDs) | ND | ND | ND | ND | ND |
| 17 | Very Severe | 6 | Normal | Outlier low  (z-score <-5 SDs) | Neg | PV/EV | ND | ND | ND |
| 18 | Very Severe | 27 | Both PEP/OI | Normal  (>-2 SDs and <+2 SDs) | Neg | ND | ND | Neg | ND |
| 19 | Very Severe | 9 | PEP | Moderate  (>-3 SDs and  <-2 SDs) | Neg | ND | ND | H. inf (PCR and Culture);  M. cat (PCR only);  S. pneu (PCR and Culture) | ND |
| 20 | Very Severe | 33 | Normal | Moderate  (>-3 SDs and  <-2 SDs) | Neg | H. inf^a^  M. cat  RSV  S. pneu Rhino | Neg | ND | ND |
| 21 | Very Severe | 32 | Normal | Severe  (>-5 SDs and  <-3 SDs) | E. col | ND | ND | ND | ND |

Abbreviations: ND, Not done; NP/OP, nasopharyngeal/oropharyngeal; PEP, primary endpoint consolidation; OI, other infiltrate; Adeno, Adenovirus; Boca, Human bocavirus; C. pneu, *Chlamydophila pneumoniae*; CMV, cytomegalovirus; HCoV, Human coronavirus; Hib, *Haemophilus influenzae* type b; Hi non-b, *Haemophilus influenzae* non-b; HMPV, Human metapneumovirus A/B; M. cat, *Moraxella catarrhalis*; Para, Parainfluenza virus; P. jirov, *Pneumocystis jirovecii*; PV/EV, Parechovirus/Enterovirus; Rhino, Rhinovirus; S. aur, *Staphylococcus aureus*; S. pneu, *Streptococcus pneumoniae*; Culture, culture; PCR, polymerase chain reaction. Lung aspirate results restricted to specimens obtained within 3 days of enrollment and those pathogens determined by the clinical review team to be non-contaminants.

^a^ Prevalence defined using NP/OP PCR density thresholds for 4 pathogens: *P. jirovecii*, 4 log_10_ copies/mL; *H. influenzae*, 5.9 log_10_ copies/mL; CMV, 4.9 log_10_ copies/mL; *S. pneumoniae*, 6.9 log_10_ copies/mL).
